# Tailored culture strategies to promote antimicrobial secondary metabolite production in *Diaporthe caliensis*: a metabolomic approach

**DOI:** 10.1186/s12934-024-02567-y

**Published:** 2024-12-05

**Authors:** Laura V. Hoyos, Luis E. Vasquez-Muñoz, Yuliana Osorio, Daniela Valencia-Revelo, Daiana Devia-Cometa, Miriam Große, Esteban Charria-Girón, Nelson H. Caicedo-Ortega

**Affiliations:** 1 Departamento de Ciencias Biológicas, Bioprocesos y Biotecnología. Facultad de Ingeniería, Diseño y Ciencias Aplicadas, Universidad Icesi, Cali, Colombia; 2 Department Microbial Drugs, Helmholtz Centre for Infection Research (HZI), German Centre for Infection Research (DZIF), Partner Site Hannover-Braunschweig, Inhoffenstrasse 7, 38124 Braunschweig, Germany; 3 Institute of Microbiology, Technische Universität Braunschweig, Spielmannstraße 7, 38106 Braunschweig, Germany; 4 Centro BioInc, Universidad Icesi, Cali, Colombia

**Keywords:** Nutrient limitation, Natural products, Antibacterial activity, Metabolomics, Submerged fermentation, Biofactory, Polyketides

## Abstract

**Background:**

In the search for new antimicrobial secondary metabolites of fungi, optimizing culture conditions remains a critical challenge, as standard laboratory approaches often result in low yields. While non-selective methods, such as modifying culture media, have been effective in expanding the chemical diversity of fungal metabolites, they have not yet established a direct link to key process parameters crucial for further optimization. This study investigates the capacity of *Diaporthe caliensis* as a biofactory for biologically active secondary metabolites, employing tailored culture media to explore the relationship between chemical diversity and critical process variables.

**Results:**

The metabolomic profiles, antibacterial activities, and production yields of the extracts were analyzed to progressively adjust the culture conditions. This study was conducted in five steps, evaluating carbon and nitrogen source concentration, nitrogen source type, salt supplementation, and pH adjustment. Altering the rice starch concentration affected biomass yield per unit of oxygen consumed, while modifications to the nitrogen source concentration influenced both the bioactivity and chemical space by *Diaporthe caliensis*. Despite changes at the metabolome level, the extracts consistently exhibited potent antibacterial activities, influenced by the nitrogen source, added salts and pH adjustments. For instance, when using corn steep liquor and rice starch, supplemented with micronutrients, different metabolites were produced depending on whether buffer or water was used, though both conditions showed similar antibacterial activities (IC_50_ ≈ 0.10 mg mL^− 1^ against *Staphylococcus aureus* and ≈ 0.14 mg mL^− 1^ against *Escherichia coli*). In the treatment where buffer was used to stabilize pH change, there was an increase in the production of phomol-like compounds which are associated with known antibiotic properties. In contrast, in the treatments using water, the drop in pH stimulated the production of previously unidentified metabolites with potential antimicrobial activity.

**Conclusions:**

This study proposes a strategic methodology for the tailored formulation of culture media aiming to promote the biosynthesis of diverse secondary metabolites. This approach revealed the critical role of nutrient limitation and pH regulation in stimulating the production of polyketide-lactone derivatives, including the antibiotic phomol. Ultimately, the systematic, custom-designed culture conditions developed in this work offer a promising strategy for expanding the chemical diversity of *Diaporthe caliensis*, while providing valuable insights into the key parameters needed for optimizing this fungal biofactory.

**Graphical Abstract:**

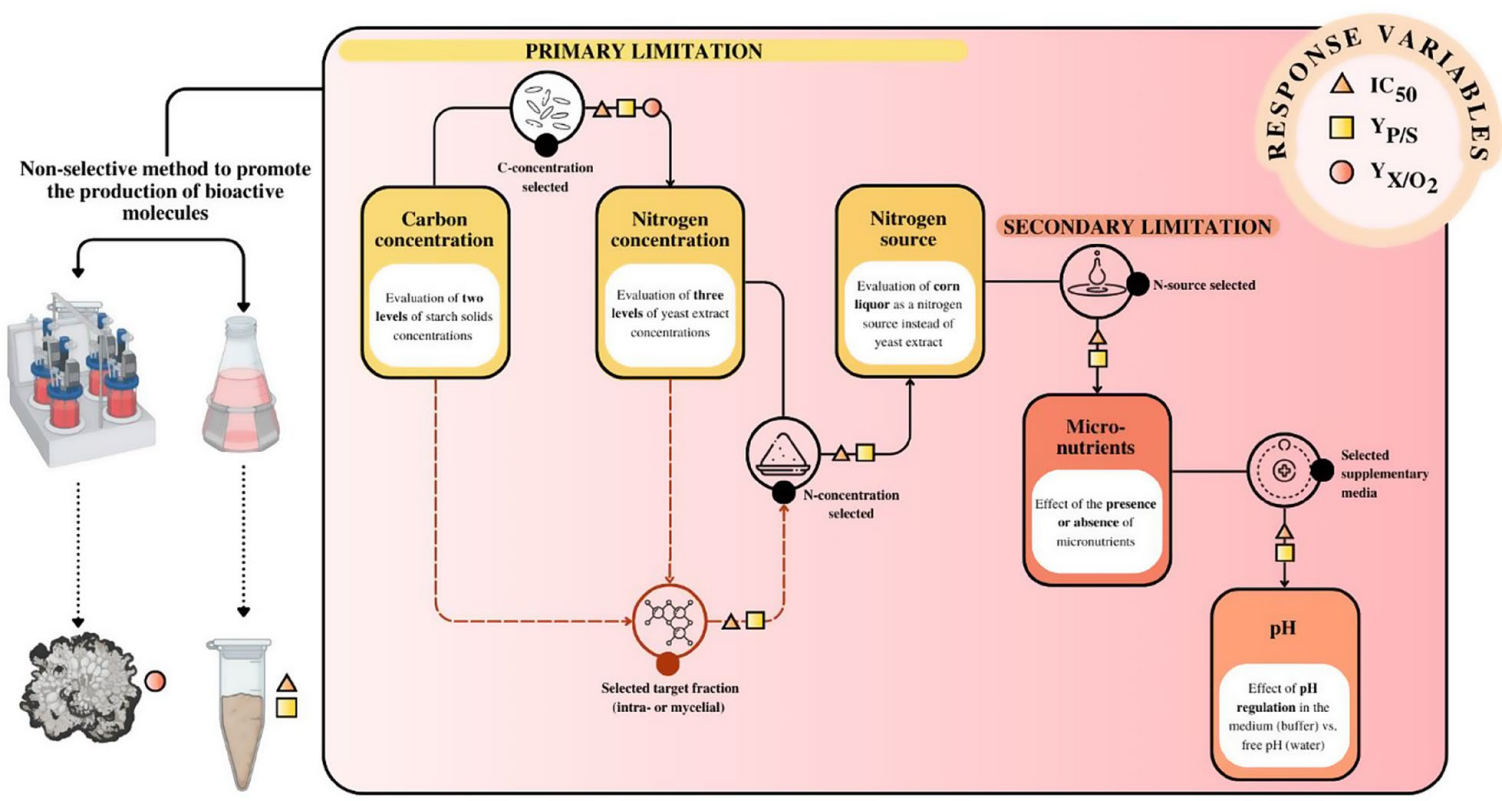

**Supplementary Information:**

The online version contains supplementary material available at 10.1186/s12934-024-02567-y.

## Background

Secondary metabolites (SMs) are low-molecular-weight compounds produced by bacteria [1, 2], fungi [3], or plants [4, 5]. Unlike primary metabolites, which are directly involved in the growth, reproduction, or development of microorganisms, SMs often facilitate and mediate microbial interactions within their environment [6]. They are involved in cell‒cell signaling, pathogenesis, and increased nutrient bioavailability [7]. In addition to conferring advantages to the producer organisms, SMs exhibit biological activities of great industrial value. SMs produced by endophytic fungi are characterized by diverse and attractive bioactivities, such as immunomodulatory [8], anticancer [9], antioxidant [10], and antimicrobial [11, 12] activities. However, traditional methodologies such as bioactivity-guided isolation face limitations, as certain molecules relevant to specific ecological niches are not produced under laboratory conditions [13]. Two main approaches are commonly used to induce their production in controlled settings: Selective and non-selective methods. The first one involves manipulating specific biosynthetic pathways using molecular biology tools, while the second one includes strategies such as the modification of culture media and fermentation conditions, offering two alternative approaches [14].

Solid-state fermentations are usually employed within non-selective methods [15, 16]. Although they offer the advantage of increased productivity [17, 18], these fermentations require large quantities of solid substrate, which negatively affects mass transfer at the substrate-fungus interface, especially oxygen diffusion [19]. In contrast, liquid cultures allow for greater mass transfer by controlling agitation and/or aeration [20]. In addition to improving the bioavailability of nutrients, it is possible to test the effect of nutritional limitations in liquid fermentation by modifying the concentrations of substrates in the medium and other culture parameters [21].

In non-selective methods, the one-strain-many compounds (OSMAC) strategy has proven effective in promoting the production of novel chemistry by modifying easily accessible cultivation parameters [22, 23]. Recent studies have explored the role of exogenous elicitors, such as chitooligosaccharides and lipids, in triggering the production of SMs in *Aspergillus fumigatus* [24]. Under nutrient-limited conditions, metabolic fluxes can shift in various organisms, favoring specific biosynthetic pathways at the expense of biomass formation [25, 26]. For example, Kottmeier et al. found that intentionally restricting phosphate increased the yield of green fluorescent protein (GFP) yield 1.87-fold in glucose-based media used to cultivate the yeast *Hansenula polymorpha* [25]. In fungi, oxygen-limiting conditions (static fermentation) can influence the production of fungal extracts with anticancer properties [9]. Another study used a coculture strategy combined with nutrient deprivation to induce cryptic biosynthetic pathways and novel antimicrobial metabolites in *Eutypa lata* and *Botryosphaeria obtusa* [27]. Furthermore, the effects of nitrogen and phosphorus limitations on the fatty acid profile produced by *Aspergillus oryzae* have been examined through transcriptome analysis [28].

When developing customized culture media to enhance the production of targeted molecules or trigger the biosynthesis of novel ones, a key challenge is integrating non-selective methods with easily measurable in situ variables, which are essential for obtaining early insights into culture performance [25]. One variable that can be monitored to identify culture patterns, such as nutrient limitations (including oxygen) or diauxic growth, is the oxygen transfer rate (OTR) in shaking flasks [29, 30]. In addition to real-time monitoring of biological variables, it is crucial to assess changes in secondary metabolite production to evaluate the effectiveness of these non-selective methods. Modifying the culture media or operating conditions can disrupt the intricate biochemical networks within fungi [31, 32]. Therefore, incorporating metabolomics analyses into bioprocess design is crucial for identifying conditions that maximize metabolite diversity and enhance the observed biological properties.

As part of our investigation into the biotechnological potential of endophytic fungi isolated from *Otoba gracilipes*, a medicinal tree native of Valle del Cauca, Colombia, we have identified promising candidates for producing laccase enzymes [33], flavors [34], antioxidant compounds [35], and secondary metabolites with antibacterial activity [36], such as phomol [37]. Inspired by these finding, and in response to the global issue of antimicrobial resistance, which was linked to 1.27 million deaths in 2019 [38–40], the aim of the present study was to design a methodology for the stepwise formulation of tailored culture media to stimulate the biosynthesis of antimicrobial metabolites in *Diaporthe caliensis*. To achieve this, we evaluated the effect of two different nitrogen sources (i.e., corn steep liquor and yeast extract) during the submerged fermentation of this fungus, using a rice starch solution as the carbon source. The effects of varying the carbon-to-nitrogen ratio were assessed in terms of (*i*) biomass yield produced per oxygen consumed (Y_X/O2_), (*ii*) OTR, (*iii*) half-maximal inhibitory concentration (IC_50_), (*iv*) yield of crude organic extracts per substrate consumed (Y_P/S_), and metabolomics data.

## Materials and methods

### Microorganisms and reagents

The endophytic fungus *Diaporthe caliensis* CM-UDEA-H27, which belongs to the fungal collection of the Universidad de Antioquia (Medellín, Colombia), was used in this study. Agar was obtained from Thermo Scientific. Malt extract, monobasic potassium phosphate, sodium tartrate dihydrate, magnesium sulfate, and iron (II) sulfate heptahydrate were obtained from Sigma‒Aldrich. Manganese (II) sulfate monohydrate from Honeywell. *D-*glucose from Scharlab. Tween 80 was obtained from Loba Chemie. Corn steep liquor (SUST PROT, lot 1866013) was obtained from Ingredion^®^. The following Colombian brand reagents were used: yeast extract (EXLV-LS^®^-3111, lot 218671) from Levapan S.A. and Caribe^®^ rice from The Diana group.

### Assessment of nutrient limitation in submerged cultivation of *Diaporthe caliensis*

A five-step experimental procedure was used to systematically evaluate the effect of primary and secondary nutrient limitations on the production of bioactive metabolites by *Diaporthe caliensis*. The strategy involved modifying one factor at a time and carefully selecting the most effective treatment before advancing to the next phase. The response variables were yield of biomass formed on a dry basis per oxygen consumed (Y_X/O2_), oxygen transfer rate (OTR), half-maximal inhibitory concentration (IC_50_ mg mL^− 1^), and yield of crude organic extracts obtained per substrate consumed (Y_P/S_). Table 1 specifies the variables used in each research step. Each experiment was performed in duplicate.

**Table 1:** Media formulations evaluated during the submerged cultivation of *Diaporthe caliensis*

Step	Treatment	Evaluated factor					Response variable					
		Rice starch solids concentration (g L^-1^)	Yeast extract concentration (g L^-1^)	Corn steep liquor concentration (g L^-1^)	Nutrient solution A	Nutrient solution B	OTR	Y_X_/_O2_ (g g^-1^)	IC_50_ (mg mL^-1^)		Y_*P*/S_ (mg g^-1^)	
									Ex	My	Ex	My
1	C25-N3.2	25	3.2	0	*X*	-	*X*	*X*	*X* ^*S*^	*X* ^*S*^	*X* ^*S*^	*X* ^*S*^
	C15-N3.2	15	3.2	0	*X*	-	*X*	*X*	*X* ^*S*^	*X* ^*S*^	*X* ^*S*^	*X* ^*S*^
2	C15-N1.5	15	1.5	0	*X*	-	*X*	*X*	*X* ^*S*^	*X* ^*S*^	*X* ^*S*^	*X* ^*S*^
	C15-N0.75	15	0.75	0	*X*	-	*X*	*X*	*X* ^*S*^	*X* ^*S*^	*X* ^*S*^	*X* ^*S*^
3	C15-N0.75-L	15	0	3.3*	*X*	*-*	-	-	*X* ^*SE*^		*X* ^*SE*^	
4	C15-N0.75-L-M (AB)	15	0.75	3.3*	*X*	*X*	-	-	*X* ^*SE*^		*X* ^*SE*^	
	C15-N0.75-L-M (B)	15	0	3.3*	*-*	*X*	*-*	*-*	*X* ^*SE*^		*X* ^*SE*^	
5	C15-N0.75-L-M-W (B)	15	0	3.3*	*-*	*X*	*-*	*-*	*X* ^*SE*^		*X* ^*SE*^	

Note: “*X*” indicates presence, and “-” indicates absence of the elements mentioned. “*” indicates a concentration equivalent to 0.75 g L^− 1^ yeast extract. “*X*^*S*^” was evaluated against *Staphylococcus aureus* “*X*^*E*^” against *Escherichia coli*. “Ex”, extracellular fraction. “My” mycelial fraction. C15-N0.75-L-M-W corresponds to the medium prepared in water

*Diaporthe caliensis* was grown in RAMOS^®^ shaking flasks to obtain the Y_X/O2_ ratio and monitor the OTR over time. These data were used to ensure shaking conditions that prevented oxygen limitation during the cultivation. After confirming this during the RAMOS^®^ experiments, the relative centrifugal force (RCF) was maintained by adjusting the speed according to the shaking diameter [41] in the Erlenmeyer flask experiments. These experiments were used to evaluate the Y_P/S_ and IC_50_ parameters using a setup strategy similar to that of Philip et al. [42]. In addition, residual sugars were quantified in both cases using HPLC to determine glucose, fructose, maltodextrin, and sucrose levels (see section ‘Chromatography’). Thus, it was confirmed that both setups had similar consumption profiles.

As a first step of this experimental approach, two different concentrations of the carbon source, formulated as the limiting nutrient, were evaluated using a rice starch solution as a substrate. This stock was prepared by heat treatment of long-grain white rice from the Colombian brand Caribe^®^. Initially, the rice grains were milled and sieved (425 μm). Subsequently, 16 g of the resulting flour was added to 400 mL of deionized water. The resulting suspension was heated in a water bath at 90 °C for 15 min.

Once the concentration of starch was chosen, the effect of two concentrations of yeast extract were tested in the second step, considering the contributions of nitrogen and carbon to the medium formulation. In the third step, the yeast extract was replaced with corn steep liquor, while maintaining the same nitrogen contribution (i.e., 90 mg L^− 1^). This concentration was based on the total nitrogen content measured using the Kjeldahl method [43]. Prior to use, the corn steep liquor was centrifuged (4 °C and 4500 rpm) to remove suspended solids, accounting for 40%, which may have included insoluble carbohydrates. The total carbon contribution was estimated following the NREL protocol [44].

Until this point (from the first to the third step), nutrient solution A (0.5 g L^− 1^ of KH_2_PO_4_ and C_4_H_4_Na_2_O_6_ ·2H_2_O) had been used to supplement the culture medium [45–48]. In the fourth step, the replacement of solution A for micronutrient solution B (0.5 g L^− 1^ of MgSO_4_ ·H_2_O, 1.0 g L^− 1^ of FeSO_4_ ·7H_2_O and 1.0 g L^− 1^ MnSO_4_ ·H_2_O) was tested according to de Souza et al. [49] as well as a combination of both solutions. For the fifth step, the influence of pH regulation using water instead of 0.05 M phosphate buffer (pH 6.3) was evaluated. Eight different treatments with varying nutritional formulations were evaluated (Table 1).

### Cultivation of *Diaporthe caliensis* in a shaking flask for online OTR assessment

The fungus *Diaporthe caliensis* CM-UDEA-H27 was cultivated on yeast malt agar (YMG) at 29 °C for seven days. Subsequently, a mycelium suspension was prepared by scraping the surface of three Petri dishes and mixing with 12 mL of 0.01% Tween 80. This suspension was used as the inoculum for the respiration activity monitoring system experiments, RAMOS^®^ (HiTec Zang GmbH, Herzogenrath, Germany). The experiments were conducted in shaking flasks with online OTR parameter measurements. After adding 1 mL of the inoculum to 49 mL of the indicated medium, the mixture was incubated at 29 °C with shaking at 140 rpm, using a 50 mm diameter and an air flow rate of 25 mL min^− 1^, with measurement cycles conducted every 30 min. The variation in OTR was monitored throughout the cultivation period. After 7 days, the biomass was separated from the supernatant by vacuum filtration through a cellulose filter paper (20–30 μm; FAST 101, Ø 12.5 cm; Chicago, Illinois, United States). The washed biomass (with deionized water) was dried using a convection oven (Binder, Germany) at 110 °C for two hours to determine the final biomass concentration (g DW L^− 1^). The presence or absence of residual sugars in the broth was confirmed through HPLC analyses (see section ‘Chromatography’). Finally, the parameter Y_X/O2_ was determined using the following equation:

1 $$\:{Y}_{X{/O}_{2}}=\:\frac{{X}_{f}}{TOT}$$

In the equation, X_f_ represents the concentration of biomass formed on a dry weight basis (g DW L^− 1^), and TOT represents the total oxygen transferred (g L^− 1^) during fermentation. This last parameter was obtained by integrating the OTR curve generated through online monitoring over cultivation time using MATLAB software (version R2023b).

### Cultivation of *Diaporthe caliensis* in an Erlenmeyer flask for secondary metabolite extraction

The inoculum preparation was adjusted to reduce the latency during cultivation. Therefore, 1.85 mL of a mycelial suspension (mycelia from one YMG agar plate mixed with 10 mL of Tween 80 solution) was added to 50 mL of YMG media prepared in 0.05 M phosphate buffer at pH 6.3 in 125 mL Erlenmeyer flasks. The cultures were incubated in an orbital shaker (Actum, Medellín, Colombia) for seven days at 90 rpm and 29 °C. The biomass was then separated by centrifugation (20 °C and 4500 rpm), washed with 0.9% v/v NaCl solution, and then disaggregated. This inoculum development had two implications: forming greater biomass and preventing pellet aggregation [50, 51].

Final fermentations were carried out in 250 mL Erlenmeyer flasks with an 80 mL working volume and 4 mL of inoculum (derived from disaggregated seed culture), keeping the relative centrifugal force (RCF) constant. Each treatment was prepared with 0.05 M phosphate buffer or water (initial pH adjusted with NaOH 8 N) at pH 6.3. For seven days, the flasks were incubated at 29 °C and shaken at 221 rpm with a 20 mm shaking diameter on a MaxQ 4450 orbital shaker (Thermo Scientific, Massachusetts, USA). The pH values were monitored from aliquots using non-bleeding pH indicator strips (McolorpH valuestat^TM;^ Merck^®^, Darmstadt, Germany).

### Secondary metabolite extraction

After cultivation, the mycelia were separated from the supernatant through centrifugation at 4500 rpm and 4 °C for 8 min, followed by two vacuum filtration steps using qualitative cellulose filters (Ø 12.5 cm, 20–30 μm), and finally washed with deionized water. The exhausted medium was concentrated using a BÜCHI R-100 rotary vacuum evaporator (BÜCHI, Flawil, Switzerland), gradually decreasing the pressure from 300 to 75 mbar at 45 °C and 100 rpm. This concentrated solution was thoroughly mixed with acetone (1:1 v/v) in an Erlenmeyer flask for 24 h at 90 rpm. Subsequently, the suspension was filtered through cellulose filters (Ø 12.5 cm, 20–30 μm) to remove precipitates. The filtrate was then concentrated to half its volume in a rotary evaporator (45 °C and 100 rpm), gradually decreasing the pressure from 650 mbar to 100 mbar. The concentrate was again extracted with ethyl acetate (EtOAc) at a 1:1 ratio (v/v) in a separating funnel at room temperature and mixed manually every 20 min for 4 h. The organic phase was concentrated to 5 mL under the same conditions and subsequently subjected to solvent removal for 24 h in an extraction cabin CEX-120 (C4, Cali, Colombia) and an additional 4 h at 45 °C in a vacuum centrifuge (Eppendorf, Hamburg, Germany).

The mycelia were macerated and extracted with acetone (1:1 w/w) for 24 h at 90 rpm. Ultrasonication was then conducted for 30 min at 45 °C, followed by filtration using a cellulose filter (Ø 12.5 cm, 20–30 μm). The mycelia were extracted again with acetone (1:1 w/w). The remaining mycelia were separated from the organic phase through filtration, and the filtrates were combined and concentrated in a rotary evaporator (i.e., 45 °C, 100 rpm, and 650 − 100 mbar). Subsequently, EtOAc was added, followed by the procedure used to obtain extracellular extracts.

Additionally, each extract was resuspended in 1 mL of methanol and mixed with the same volume of *n-*heptane. The mixture was shaken in a MultiTherm shaker (Benchmark Scientific, Sayreville, USA) at 300 rpm and 25 °C for 2 h to facilitate phase contact. The methanolic phase was recovered and dried in a gas extraction cabinet (24 h) and a vacuum centrifuge (4 h, 45 °C). From the mass of the crude and dry extracts obtained, the Y_P/S_ was calculated by the following Eq. (2):

2 $$\:{Y}_{P/S}=\frac{{m}_{e}}{{m}_{s}}$$

where m_e_ corresponds to the mass (mg) of crude and dry extract and m_s_ refers to the mass (g) of starch solids added to the initial medium.

### Antibacterial activity assays

The antibacterial activity of the crude extracts was determined following the methodology described by Charria-Girón et al. [36]. Initially, the extracts were evaluated against *Staphylococcus aureus* ATCC 25,923, and those with the best IC_50_ values were subsequently tested against *Escherichia coli* ATCC 25,922. Bacterial cultures were prepared by taking 100 µL of each strain preserved in a cryovial and adding it to a flask with 50 mL of Luria–Bertani (LB) broth at pH 7.0. After 20 h of incubation (37 °C and 150 rpm), the culture was sampled and seeded on an LB agar plate. After another 20 h at 37 °C, four colonies were taken to prepare a bacterial suspension using 0.9% (v/v) saline solution (OD_620_ nm between 0.08 and 0.1). According to the McFarland scale, the estimated cell concentration was 1 × 10^8^ CFU mL^− 1^, so dilutions were performed to reach a concentration of 1 × 10^4^ CFU mL^− 1^. The obtained crude extracts were evaluated at concentrations ranging from 2 to 10 mg mL^− 1^ and were dissolved in a mixture of 20% methanol and 80% DMSO (1% v/v). Measurements were performed on six serial dilutions in triplicate, with LB broth and methanol-DMSO (1% v/v) serving as negative controls. The assay plates were incubated for 20 to 24 h at 37 °C and 140 rpm before the OD_620_ was measured using a spectrophotometric reader (Thermo Fisher Scientific™ Varioskan™ LUX, United States).

## Chromatography

### Detection of residual sugars

Samples of cell-free medium (supernatant) were filtered using a 0.2 μm cellulose filter, and the pH was adjusted to between 1 and 6 using 5 mM sulfuric acid. The presence of residual sugars was then detected by HPLC analysis (Thermo Fisher Scientific, Waltham, MA, USA) using an Aminex HPX-87 H column (hydrogen-form, 300 × 7.8 mm, from Bio-Rad) and an RI detector. This analysis used a sulfuric acid solution (5 mM) as the mobile phase with a flow rate of 0.6 mL min^− 1^ and an operating temperature of 45 °C. Five solutions with different concentrations of glucose, fructose, maltodextrin, and sucrose were injected as standards. These results are not shown in the article since they correspond to a comparison of the type of nutritional limitation achieved in both configurations (RAMOS and Erlenmeyer flasks).

### Metabolomics

The crude extracts were dissolved in acetone: methanol (1:1 v/v) to a concentration of 4.5 mg mL^− 1^ for subsequent analysis via LC/MS. The measurements were recorded using an UltiMate 3000 Series UHPLC (Thermo Fischer Scientific, Waltham, MA, USA) with a C18 Acquity UPLC BEH column (2.1 × 50 mm, 1.7 μm; Waters, Milford, MO, USA) connected to an AmaZon speed ESI-Iontrap-MS (Bruker Daltonics, Bremen, Germany). The analyses were performed using a 0.6 mL min^− 1^ flow rate, an injection volume of 2 µL, and 40 °C. For the mobile phase, H_2_O + 0.1% FA (Solvent A) and ACN + 0.1% FA (Solvent B) were used with the following gradient: 5% B for 0.5 min, 5–100% B for 20 min and 100% B for 4.5 min. The UV/Vis data were recorded using a Diode Array Detector (DAD) between 190 and 600 nm, and the results were analyzed using the software Data Analysis 4.4 (Bruker Daltonics, Bremen, Germany).

The concentration of each sample was measured using the instrumental strings and conditions reported by [51] for metabolomic analyses. The raw data were preprocessed with a MetaboScape 2022 (Bruker Daltonics, Bremen, Germany) in the retention time range of 1.0–20 min, and the obtained features were dereplicated based on their accurate molecular weight and MS/MS spectra against the compounds as previously reported for the genus *Diaporthe* or *Phomopsis* in the Natural Product Atlas (NP Atlas) database [52]. For this purpose, MetaboScape automatically performed in silico MS/MS matching based on the InChI-encoded structures using the MetFrag algorithm without MS/MS reference data [53]. Subsequent analyses were performed using R and RStudio software (version 4.2.1).

### Data processing and statistical analysis

Determination of IC_50_ values was conducted using R and RStudio software (version 4.2.1) with the ggplot2 and drc packages fitted to a four-parameter model. Hierarchical cluster analysis was performed using the Ggdendro package. Statistically significant differences in production yields and IC_50_ values among the various treatments were analyzed using MINITAB^®^ 19 statistical software. An ANOVA was performed to assess the variation in production yields and IC_50_ values.

## Results

The effect of nutrient limitations (i.e., carbon, nitrogen, and micronutrients) on the production of secondary metabolites during the fermentation of *Diaporthe caliensis* was assessed through the evaluation of three response variables: Y_P/S_, IC_50_, and Y_X/O2_. The results are presented in two stages, where primary and secondary substrates are studied by the systematic formulation of culture media.

### Assessment of nutrient limitation in submerged cultivation of *Diaporthe caliensis*: primary limitation

**Fig. 1:**
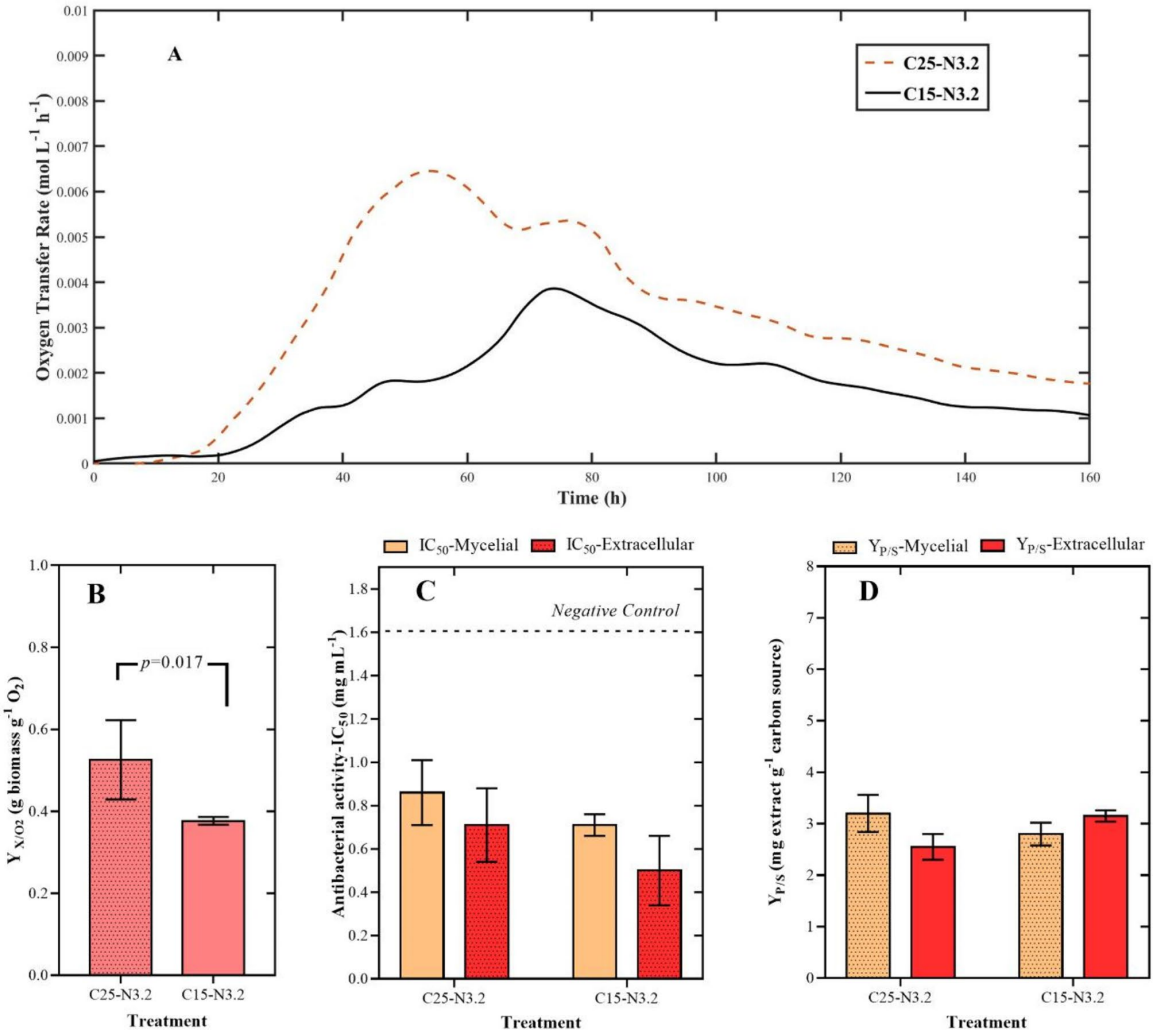
Effect of carbon source concentration (rice starch) on the performance of a batch culture of *Diaporthe caliensis* and its respective crude extracts: C15-N3.2 and C25-N3.2 treatments. **(A)** Changes in OTR (mol L^− 1^ h^− 1^) over time for each treatment. **(B)** Calculated Y_X/O2_ (g biomass g-1 O_2_) for each treatment. **(C)** Antibacterial activity (IC_50_ in mg mL^− 1^) evaluated against *Staphylococcus aureus* (ATCC 25923*).* Statistically insignificant variable (*p* value > 0.05). **(D)** Calculated Y_P/S_ (mg extract g-1 carbon source) for mycelial and extracellular extracts. *Statistically insignificant variable (p value > 0.05).* The antibacterial activities of the strains were contrasted with that of the negative control (black dotted line). Each value represents the mean of two biological replicates (*n* = 2). Cultivation conditions: **(A)** RAMOS^®^ device (250 mL unbaffled shake flask with 50 mL of medium, shaking diameter of 50 mm, and shaking frequency of 140 rpm); **(B)** and **D)** orbital shaker (250 mL unbaffled shake flask with 80 mL of medium, shaking diameter of 20 mm and shaking frequency of 221 rpm)

The impact of carbon limitation on the production of secondary metabolites by *Diaporthe caliensis* was investigated by evaluating two different concentrations of a rice starch solution as a substrate (15 and 25 g L^− 1^ starch). Figure 1A shows the oxygen transfer rate (OTR) curves for the two treatments, indicating no oxygen limitation throughout the cultivation period and consumption of more than one substrate, according to [54]. Online OTR measurements also revealed a delayed growth phase between 15 and 20 h for each treatment. While the C15-N3.2 treatment lacked the typical exponential increase (74 h for OTR max), the C25-N3.2 treatment reached the maximum peak at 53 h. The lower slope for the C15-N3.5 treatment may be associated with a metabolic difference in starch hydrolysis dynamics. In both cases, an OTR value of zero was not reached, probably due to the cell maintenance condition. Overall, lower total oxygen consumption was observed for the experiment with a low carbon source concentration.

Fig. 1B summarizes the biomass-oxygen yields for each treatment, indicating that a higher starch concentration led to increased biomass formation at the expense of transferred oxygen (0.53 g biomass g^− 1^ O_2_ ± 0.1). Figure 1C and D present the antimicrobial activity (IC_50_) and Y_P/S_ results for the mycelial and extracellular extracts, respectively, even though neither variable was significant (*p* value > 0.05). Notably, a lower biomass-oxygen yield (Y_X/O2_) might result in greater secondary metabolite production. Therefore, 15 g L^− 1^ of the starch solution was chosen for further experiments (corresponding to treatment C15-N3.2)

**Fig. 2:**
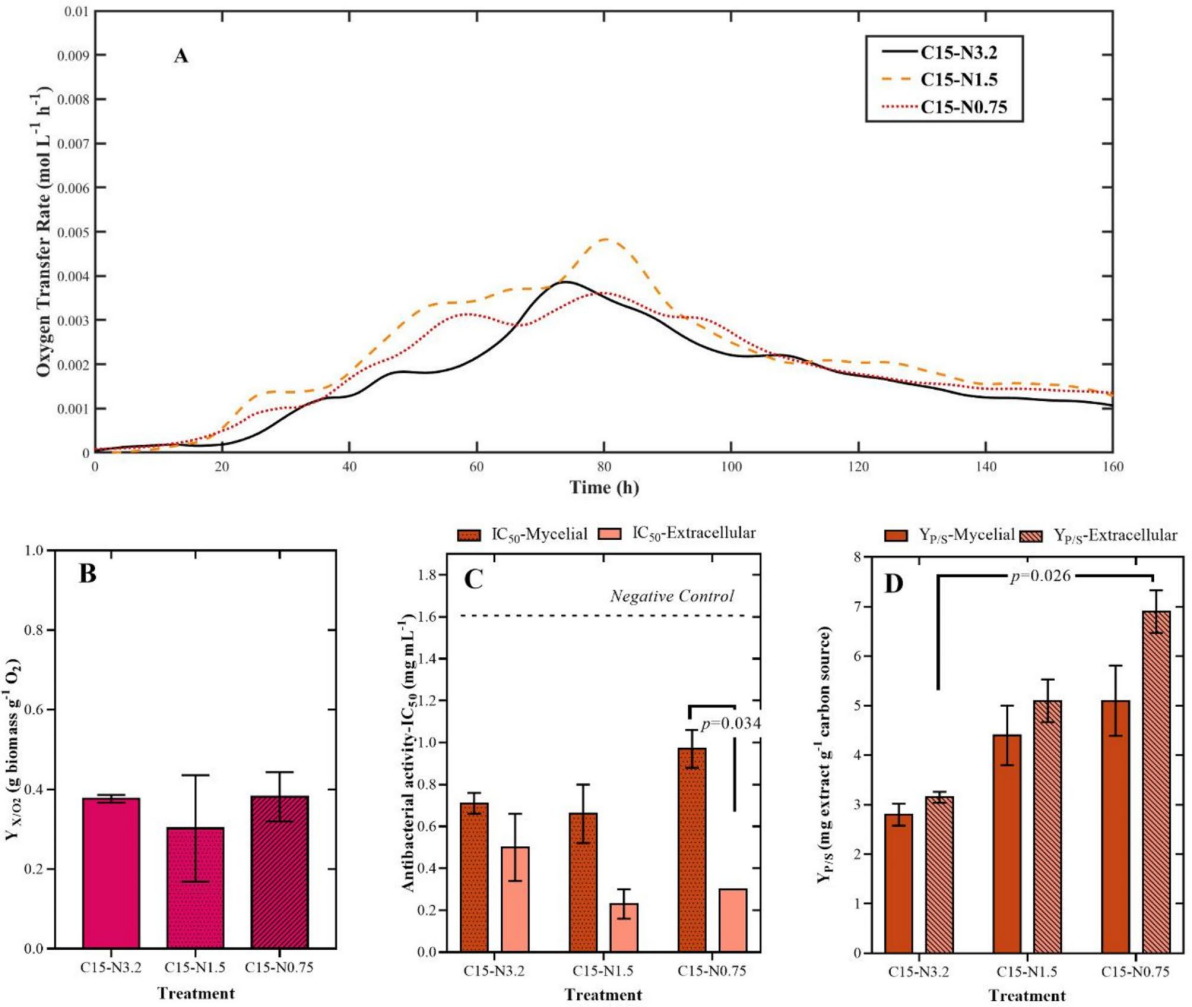
Effects of different concentrations of nitrogen source (yeast extract) on the performance of a batch culture of *Diaporthe caliensis* and its respective crude extracts: C15-N3.2, C15-N1.5, and C25-N0.75 treatments. **(A)** Changes in the oxygen transfer rate (OTR, mol/L/h) over time for each treatment. **(B)** Biomass-oxygen yield (Y_X/O2_, g biomass g^− 1^ O_2_) for each treatment. *Statistically insignificant variable (p value > 0.05).***(C)** Antibacterial activity (IC_50_ in mg mL-1) evaluated. *Staphylococcus aureus* (ATCC 25923). **(D)** Crude extract-substrate yield (Y_P/S_, mg extract g-1 carbon source) for mycelial and extracellular extracts. The antibacterial activities of the strains were contrasted with that of the negative control (black dotted line). Each value represents the mean of two replicates (*n* = 2). Cultivation conditions: **(A)** RAMOS^®^ device (250 mL unbaffled shake flask with 50 mL of medium, shaking diameter of 50 mm, and shaking frequency of 140 rpm); **(B)** and **(D)** orbital shaker (250 mL unbaffled shake flask with 80 mL of medium, shaking diameter of 20 mm and shaking frequency of 221 rpm)

During the second step, yeast extract was tested as the sole nitrogen source at three different concentrations, while the starch concentration was kept at 15 g L^− 1^. Figure 2A illustrates that, compared to those of the carbon-limiting experiments, the OTR curves of these experiments were very similar. After 16 h of fermentation, both the C15-N0.75 and C15-N1.5 treatments started their exponential growth phase and reached the maximum OTR values after 80 h. Similarly, changes in Y_X/O2_ were not statistically significant (Fig. 2B). In contrast, significant differences were found in some treatments for the Y_P/S_ and IC_50_ variables. In general, the crude extract yield increased with decreasing concentrations of yeast extract (Fig. 2D). In all the treatments, the extracellular extracts had higher yields. The highest Y_P/S_ value (6.9 mg of crude extract per g of starch ± 0.43) was obtained for the C15-N0.75 treatment.

For antibacterial activity (Fig. 2C), the mycelial extracts demonstrated less desirable IC_50_ values. The lowest IC_50_ value was 0.66 mg mL^− 1^ ± 0.14, which was greater than the 0.3 mg mL^− 1^ ± 0.0 obtained from the same treatment (C15-N1.5). Notably, a more pronounced difference between the fractions was observed for the C15-N0.75 treatment, with IC_50_ values of 0.97 mg mL^− 1^ ± 0.09 and 0.3 mg mL^− 1^ ± 0.0 for the mycelial and extracellular extracts, respectively. Based on these findings, it was determined that *(i)* the extracellular extract was of greater interest, as the antibacterial activity of the mycelial extract was generally lower across all treatments. *(ii)* Treatment C15-N0.75 was chosen as the starting point for subsequent experiments due to its highest Y_P/S_. In addition, *(iii)* for the subsequent steps, the variable Y_X/O2_ was not considered, as it was insignificant in the context of the nitrogen limitation experiments.

**Fig. 3:**
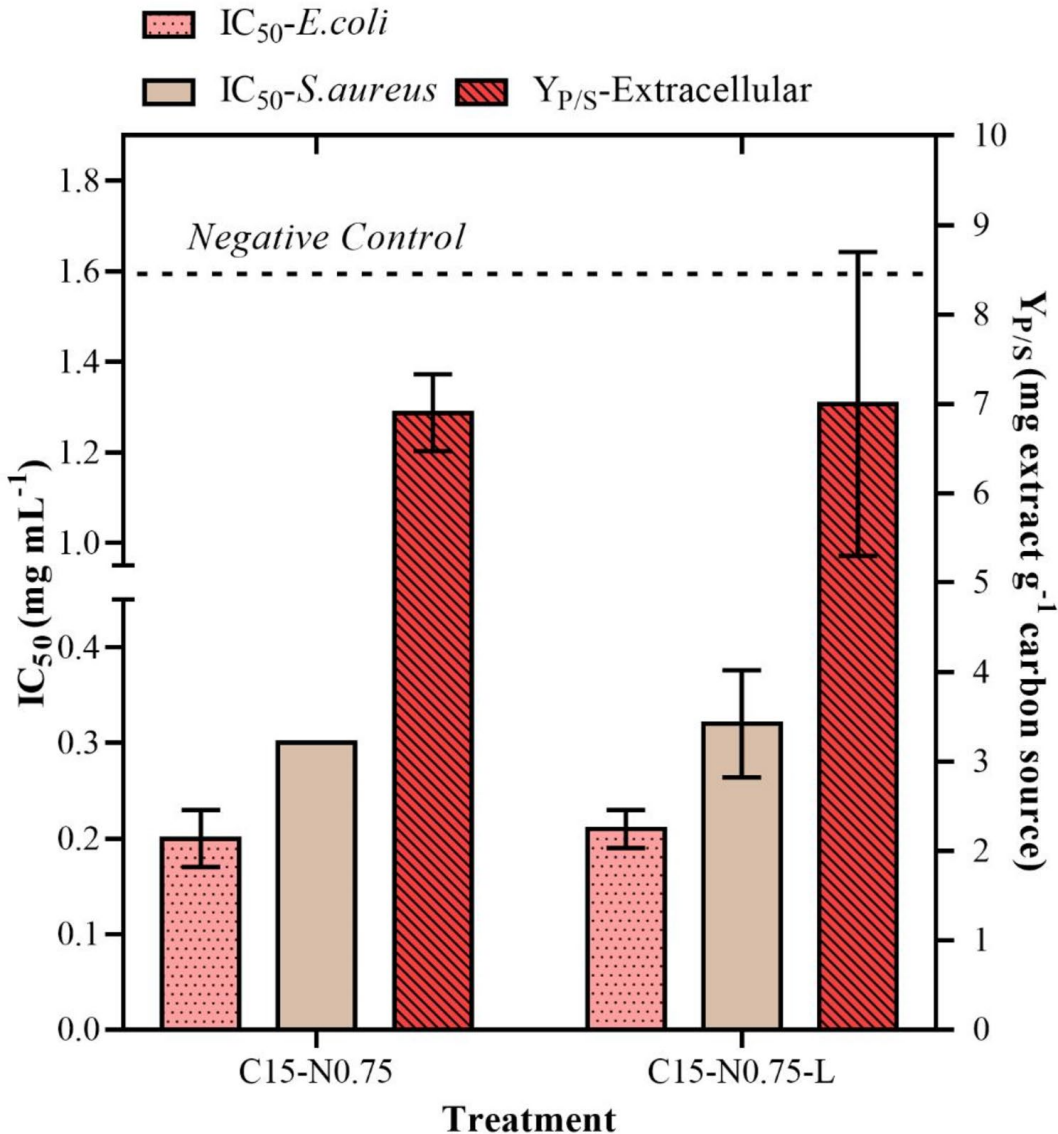
Effects of nitrogen sources (i.e., yeast extract, C15-N0.75; and corn liquor, C15-N0.75-L) on antibacterial activity and yield of crude extracts obtained from batch culture of *Diaporthe caliensis*: Antibacterial activity (IC_50_ in mg mL^− 1^) evaluated against *Staphylococcus aureus* (ATCC 25923) and *Escherichia coli* (ATCC 25922). *Statistically insignificant variable (p value > 0.05).* Moreover, the product-substrate yield (Y_P/S_, mg extract g^− 1^ carbon source) was calculated for the extracellular extracts. *Statistically insignificant variable (p value > 0.05)*

The effect of the type of nitrogen source was assessed by replacing yeast extract with corn steep liquor, using the C15-N0.75 treatment as a reference. The performance of each treatment was evaluated by comparing the Y_P/S_ and IC_50_ values against two reference bacteria (*Staphylococcus aureus* ATCC 25923 and *Escherichia coli* ATCC 25922) as the response variables. When using corn steep liquor, Y_P/S_ was 5.7 mg of extract per g of carbon source, which was lower than that when using yeast extract (6.9 mg of crude extract per g of starch ± 0.43) (Fig. 3). No clear differences in the IC_50_ were detected between the treatments for either bacterial strain (0.21 mg mL^− 1^ ± 0.02 for *Escherichia coli* and 0.32 mg mL^− 1^ ± 0.056 for *Staphylococcus aureus*). Although the findings were not statistically significant, we decided to continue experimenting with corn steep liquor (C15-N0.75-L) because it is a low-cost source of bioactive secondary metabolites that has been relatively unexplored.

### Assessment of nutrient limitation in submerged cultivation of *Diaporthe caliensis*: secondary limitation

For the treatments with corn steep liquor as a nitrogen source (C15-N0.75-L), there were some medium modifications through the addition or replacement of nutrients contained initially in solution A (Table 1): C15-N0.75-L-M (AB) and C15-N0.75-L-M (B).

**Fig. 4:**
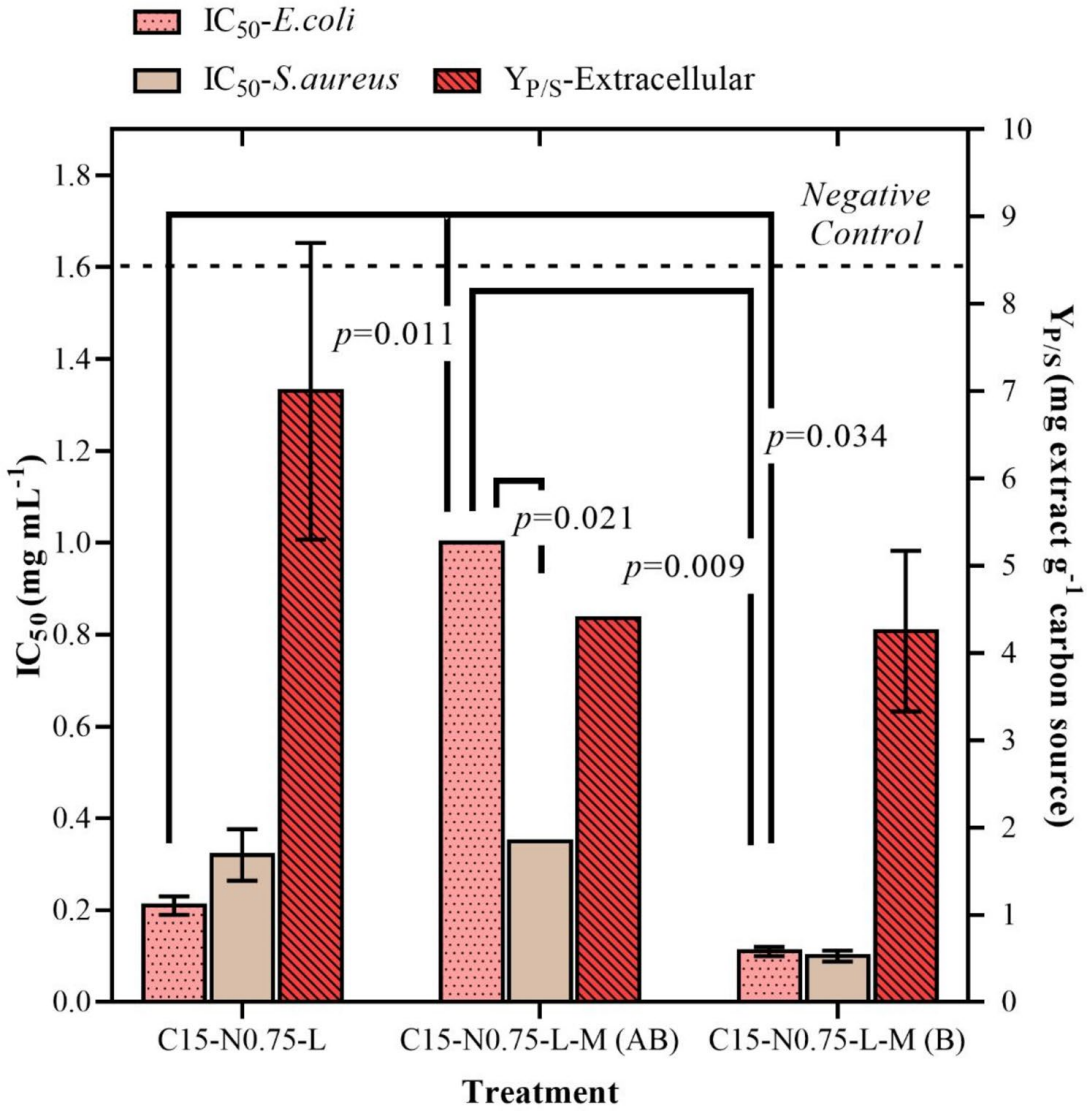
Effect of micronutrient addition (treatments C15-N0.75-L, C15-N0.75-L-M, and C15-N0.75-L-M-W) on the antibacterial activity and yield of crude extracts obtained from batch culture of *Diaporthe caliensis*: Antibacterial activity (IC_50_ in mg mL^− 1^), evaluated against *Staphylococcus aureus* (ATCC 25923) and *Escherichia coli* (ATCC 25922) and product-substrate yield (Y_P/S_, mg extract g^− 1^ carbon source) for extracellular extracts

The IC_50_ values for *Staphylococcus aureus* improved with micronutrient replacement. The antibacterial activity decreased from 0.32 mg mL^− 1^ ± 0.56 to 0.10 mg mL^− 1^ ± 0.06 when the salt composition was modified (i.e., MgSO_4_·H_2_O, FeSO_4_·7H_2_O and MnSO_4_·H2O were added in place of KH_2_PO_4_ and C_4_H_4_Na_2_O_6_·2H_2_O). A similar result was observed for gram-negative *Escherichia coli*. However, the Y_P/S_ yield decreased to 4.25 mg of extract per g of starch ± 0.46 (25.44%). Moreover, the combination of all the micronutrients negatively affected the activity of the extracts against *Escherichia coli* (0.93 mg mL^− 1^ ± 0.11), and a moderate decrease in the Y_P/S_ was observed. These results are presented in Fig. 4.

**Fig. 5:**
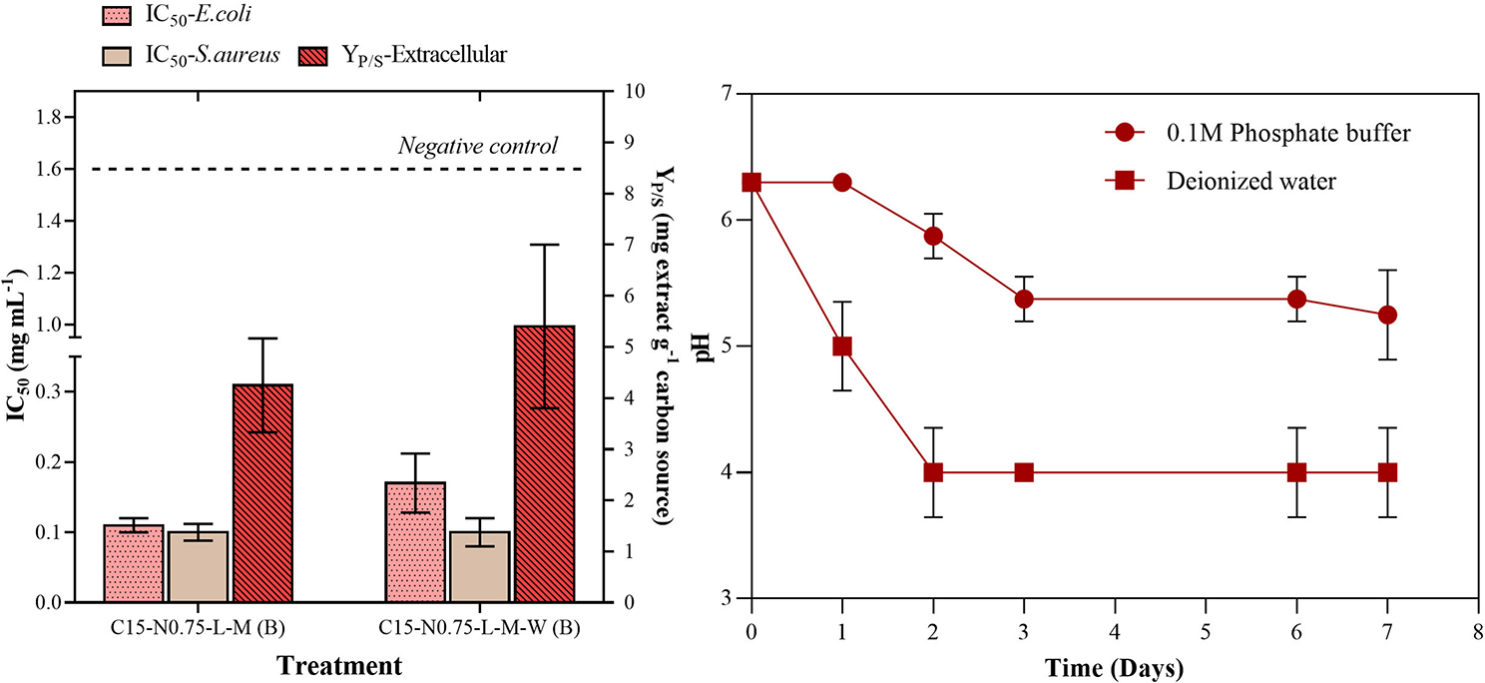
Effect of pH on the performance of a submerged batch culture of *Diaporthe caliensis* and their respective crude extracts: C15-N0.75-L-M (buffered) and C15-N0.75-L-M-W (not buffered) treatments. (A) Antibacterial activity (IC_50_ in mg mL^− 1^) of the extracellular extracts evaluated against *Staphylococcus aureus* (ATCC 25923) and *Escherichia coli* (ATCC 25922) and product-substrate yield (Y_P/S_, mg extract g^− 1^ carbon source). *Statistically insignificant variables (p value > 0.05)* were also included. (B) pH culture value over time (mean value of two replicates)

The C15-N0.75-L-M treatment was compared to the C15-N0.75-L-M-W treatment, which had the same formulation but without pH buffering. The latter treatment was prepared in water, and the pH was adjusted only at the beginning of fermentation. The antibacterial activity against *Staphylococcus aureus* remained constant, but Y_P/S_ showed an improvement of 5.40 mg of crude extract per g of starch ± 0.80. These results are displayed in Fig. 5A. Conversely, monitoring the pH (Fig. 5B) of the medium during the cultivation period revealed that the application of phosphate buffer stabilized the pH at 6.3 during the initial incubation time, and a constant value of approximately 5.5 was maintained after the third day. In contrast, the nonbuffered treatment decreased the biological activity, resulting in a pH of 4.0 starting from the second day of cultivation.

### Metabolomic analysis

**Fig. 6:**
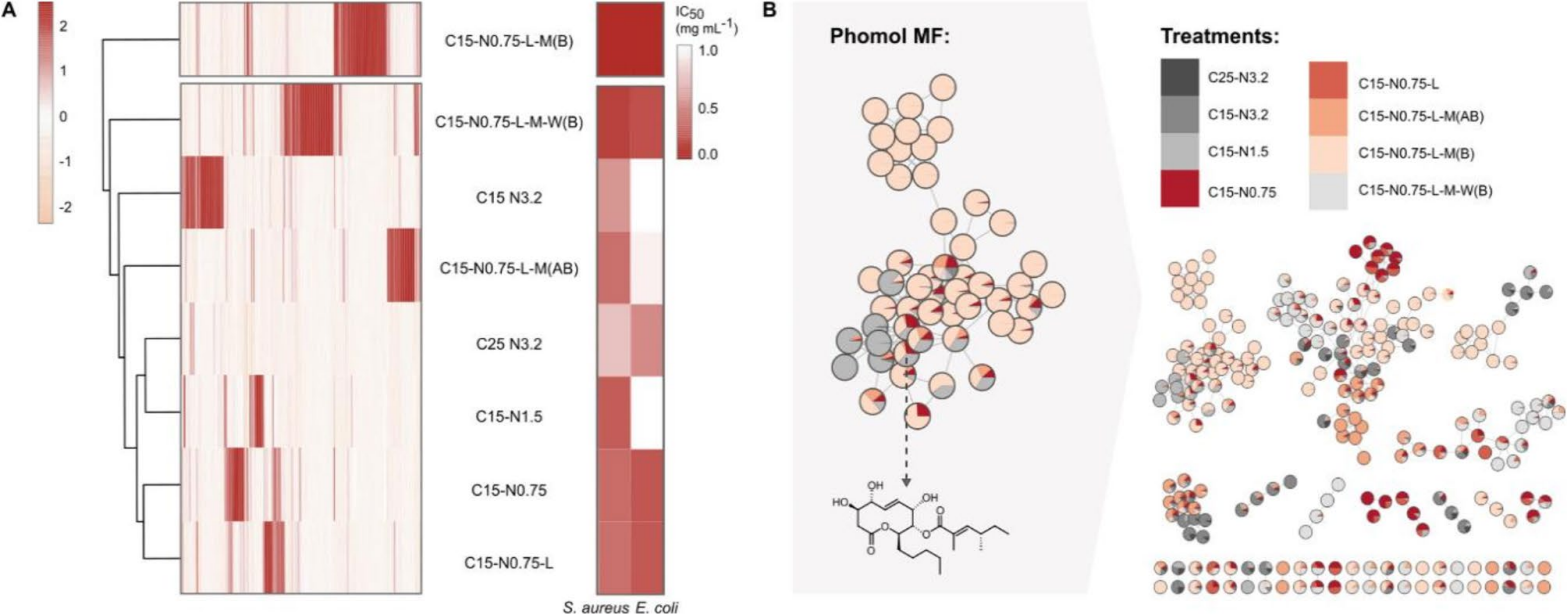
**(A)** Heatmap following a hierarchical clustering of MS features detected in the extracellular crude extracts of all treatments derived from the cultivation of *Diaporthe caliensis* (left) using the complete linkage method and the Euclidean distance metric. The heatmap displays feature abundance values with hierarchical clustering of features and crude extracts obtained from different media. The scaled and centered abundances are color coded from red (high abundance) to light orange (low abundance). Heatmap of the antibacterial activities (IC_50_ in mg mL^− 1^) of the extracellular crude extracts of all the treatments evaluated against *Staphylococcus aureus* (ATCC 25923) and *Escherichia coli* (ATCC 25922) (right). A heatmap with dendrograms was generated with the R package pheatmap. **(B)** Feature-based molecular networking (FBMN) plot of the compounds detected in all the treatment groups (right), with pie charts representing the treatments in which the spectra of the compounds were detected. The Molecular Family (MF) encompassing phomol-like features (left)

To evaluate the effect of media formulation on secondary metabolite production by *Diaporthe caliensis* during submerged cultivation in shaking flasks, all 7415 MS features detected in all the treatments were grouped by hierarchical clustering (HCA) after blank features removal. The data were centered and scaled during preprocessing, resulting in normalized abundance values that indicate features with lower or higher intensities relative to the mean. These standardized values are represented on the x-axis of the heatmap (Fig. 6A). Notably, treatment pairs such as C25-N3.2 and C15-N1.5, as well as C15-N0.75 and C15-N0.75-L, clustered together, indicating similarities in their metabolomes. Conversely, the remaining treatments formed distinct clades, highlighting substantial metabolome differences. Notably, these metabolomic patterns did not directly correlate with the antibacterial properties against the evaluated bacterial strains (i.e.,* Staphylococcus aureus* and *Escherichia coli*). In fact, despite the metabolome being markedly altered in most cases, the antibacterial activities (IC_50_) remained similar.

To further inspect the changes at the metabolome level depending on the media formulation, a feature-based molecular network (FBMN) was constructed. The analysis revealed that 237 features were grouped into 35 molecular families (MFs) with at least two clustered nodes (Fig. 6B) and 2670 singletons. The molecular families were differentially distributed across the different treatments, even though a reduced chemical space was observed for the C25-N3.2 treatment compared to the other treatments. A decision was made to explore the chemical diversity within the obtained crude extracts by using CANOPUS to predict the respective natural product classes de novo based on the MS/MS spectra [55]. This analysis determined that the most abundant class of compounds corresponded to polyketides, although revealing a high chemical diversity within the major MFs. Accordingly, their respective features were classified as alkaloids, amino acids and peptides, carbohydrates, fatty acids, polyketides, shikimates, phenylpropanoids, terpenoids, and even some without annotation (Additional file 1). Out of the 2907 MS/MS features detected, only 39 were annotated, and 5 were traced back to different MFs (Additional file 2, Table 1), accounting for just 0.5% of total MS features and 1.3% at the MS/MS level.

After dereplication, phomol was identified and found to be absent in the C25-N3.2 treatment, but it appeared once the carbon and nitrogen contents were reduced, indicating a positive relationship between nitrogen limitation and phomol-like molecule production. However, phomol-like molecules were not detected in the C15-N0.75-L-M-W (B) treatment, which differed only by the absence of buffer, leading to differences in chemical space and pH by the end of fermentation. Accordingly, the highest production of phomol-like molecules was observed in the C15-N0.75-L-M (B) treatment, as shown in Fig. 6B, which also demonstrated higher production titers of phomol and its related derivatives compared to their production in solid oat medium, as previously reported (Additional file 2, Fig. S2) [37]. For phomodiol and chaetoaurin, which were grouped within MFs comprising 14 and 3 nodes respectively, a similar trend to phomol-like molecules was observed, showing higher abundance in the C15-N0.75-L-M (B) treatment. However, some features within the phomodiol MF appeared to be more prominent in treatments with higher carbon and nitrogen content. Meanwhile, phomophyllin D and E were produced widely across different treatments, though our FBMN analysis suggests that more complex terpenoid derivatives were almost exclusively produced in the C15-N0.75-L-M-W (B) treatment. Nevertheless, it is of utmost interest that the major MF in our FBMN analysis remains entirely unannotated. However, CANOPUS results suggest that the features within this MF are likely to be chemically diverse, indicating that the main SMs of *Diaporthe caliensis* are yet to be discovered.

Overall, both the HCA and FBMN analyses showed similar results, illustrating that the chemical space produced by *Diaporthe caliensis* was tailored by modifications in the culture medium. The highest number of MS/MS features was detected in cultures with a lower nitrogen content and supplemented with micronutrients. In addition, a greater chemical diversity was observed when the nitrogen content was reduced to its lowest level, resulting in a much richer metabolome beyond the previously identified polyketide-lactones produced by this fungus. Given the fact that the observed changes in the antibacterial effects for the different treatments did not directly correlate with the production of phomol-related molecules, which were confined to a limited number of treatments, it is expected that other unidentified molecules are responsible for these biological effects. The results of this study underscore that the chemical diversity produced by *Diaporthe caliensis* remains largely uncharted when compared to the known secondary metabolites of this genus, suggesting the potential for discovering other bioactive molecules beyond polyketide-lactones related to phomol.

## Discussion

This work focused on the formulation of tailored media to improve the production of bioactive SMs during the cultivation of *Diaporthe caliensis*. Fungi from this genus are known for producing secondary metabolites with diverse biological activities, such as anti-inflammatory, antibacterial, cytotoxic, and neuroprotective effects [31]. Despite the vast chemical diversity within the genus *Diaporthe*, to the best of our knowledge, no systematic study has been reported on the design of tailor-made media to improve the production of bioactive molecules. Therefore, this study proposes a five-step approach for selecting an optimal culture medium to yield crude extracts with antibacterial activity.

The results presented herein suggested two trends: *(i)* primary metabolism was predominantly affected when the carbon source concentration was modified, and *(ii)* altering the nitrogen source concentration (i.e., yeast extract) principally impacted secondary metabolite production. This observation is supported by monitoring changes on the Y_X/O2_, IC_50_, and Y_P/S_ across the different treatments. Specifically, during the first step of this study, it was found that the higher concentrations of the starch solution led to an increased Y_X/O2_ ratio and greater biomass formation (Additional file 3). In addition, the OTR curves (markedly Fig. 1A) indicated a tendency to consume multiple substrates, likely originating from both enzymatic transformation and thermal pretreatment, during which starch is fractionated into dextrins and other simple sugars, thus leaving more than one substrate available. It is well understood that rapidly assimilable carbon sources are preferentially utilized during growth [56, 57]. Once depleted, more complex substrates are consumed, initiating the idiophase [57]. Consequently, a higher carbon source concentration delays the onset of the phase in which SMs are produced [58]. Therefore, the antibacterial activity, Y_P/S_ yield, and metabolomic profiles of the extracts from the C15-N3.2 and C25-N3.2 treatments did not differ significantly.

However, when the effect of modifying the nitrogen source concentration was studied, it was found that lower levels of yeast extract resulted in greater yields of crude extract (Y_P/S_). Although the antibacterial activity did not substantially improve when the concentration decreased from 3.2 g L^− 1^ to 0.75 g L^− 1^ nitrogen, a greater diversity of molecules was promoted, according to our metabolomics analyses. Generally, fungi respond to changes in nitrogen availability through very complex regulatory mechanisms [59]. For instance, *Aspergillus* spp. are known to produce SMs of interest (i.e., aflatoxin, ochratoxin, orsellinic acid, and spiroanthrones) when subjected to conditions of nitrogen repression or starvation [60–62]. It is possible that reduced nitrogen availability may accelerate the initiation of the idiophase, during which SMs are typically produced, as microorganisms can be sensitive to their own products during the trophophase [56].

The concentrations of carbon and nitrogen sources were first chosen to limit one nutrient over the other. For rice starch, the polymer content was measured by Lugol’s iodine staining, while the sugar content was determined by HPLC to verify the experimental limitations. However, for nitrogen, no strategy was established to measure its content at the end of fermentation without interference from proteins or peptides secreted by the fungus. As a result, it could not be robustly confirmed whether nitrogen limitation was achieved in some of the treatments. As an indirect alternative, simple sugars were present in the C15-N0.75 treatment (see Additional file 4), suggesting that nitrogen could have been depleted by the end of the fermentation period. These limitations highlight the need for future research employing more sensitive methods to confirm the nutritional conditions to which the fungus is exposed.

In addition to the nitrogen concentration, the effect of changing the nitrogen source by substituting corn steep liquor with yeast extract was evaluated. The preliminary choice of corn steep liquor was because although it is a vastly available resource, it has not been frequently used in evaluating SM production [63]. However, any change in complex sources in the culture medium must be evaluated because different nutritional compositions can significantly influence metabolic pathways [64] and physiological and morphological responses [59, 65]. Although no significant differences in the IC_50_, Y_P/S,_ or metabolome were found in this study, there was a change in the relative intensity of the phomol-like molecules (Additional file 5). This effect of the nitrogen source on the production of a particular group of molecules has been reported for *Y. lipolytica*, where the substitution of yeast extract for corn steep liquor led to an increase in intracellular enzymes [63]. This phenomenon was explained by increased activity in the pyruvate carboxylation pathway, which suggested a redistribution of carbon flux [63].

Furthermore, we also evaluated the addition of two different supplementary solutions. The media containing solution B exhibited improved IC_50_ values compared to those with solution A alone (see Materials and Methods section) or a combination of both solutions. The magnesium in solution B is crucial for protein synthesis [66], while iron is essential for transcription, replication, and energy production [67]. Although the exact function of manganese is poorly understood, its significance in fungal homeostasis is well known [68]. Sodium and potassium are vital for maintaining osmotic balance, generating membrane potential, and regulating ion or molecule transport [69]. While both solutions effectively promoted the production of antibacterial compounds, their combinations might have induced osmotic stress, leading to observable changes in the metabolome (Fig. 6A) and alterations in antibacterial activity, as evidenced by lower IC_50_ values against *Escherichia coli* observed with treatments supplemented with solutions A and B.

In the last step of this study, treatments were carried out with 0.05 M phosphate buffer and water. In both cases, the pH tended to decrease due to fungal metabolic activity. However, this change was much more pronounced in the absence of buffer. Although neither the IC_50_ nor the Y_P/S_ ratio reflected these differences, the metabolomic profiles were markedly different. For instance, the production of phomol-like molecules was greater in the C15-N0.75-L-M (B) buffer treatment (Additional file 5). This could be attributed to *(i)* the availability and consumption of nutrients, which depend on the pH of the medium, particularly for the absorption of iron [67], a component of solution B. Thus, different biosynthetic pathways might be stimulated, as some substrates are more readily utilized in one treatment compared to others. On the other hand, *(ii)* it is possible that the production of molecules such as phomol is pH dependent, similar to the polyketides hibarimicins A-G [70]. Although different molecules were produced, the crude extracts exhibited similar bioactivities. Thus, rather than focusing on a specific class of molecules, this metabolomics-guided strategy highlights how variations in the culture conditions of *Diaporthe caliensis* can alter its metabolome and enhance desirable biological activities. Our in-depth metabolomics analysis demonstrated that the proposed methodology effectively expanded the chemical diversity of *Diaporthe caliensis*, prompting the production of SMs besides the previously associated polyketide-lactones known from this fungus. Moreover, despite our dereplication strategy relying on in-silico annotation based on mass accuracy, isotopic pattern, and MS/MS spectra, the combination of different approaches revealed a high chemical diversity across all treatments. The putative annotation of chaetoaurin, phomodiol, and phomophyllins, whose presumed biosynthetic origins were in agreement with the CANOPUS predictions further support this hypothesis [71–74]. In fact, these SMs are reported to exhibit diverse biological activities, with phomophyllins, in particular, belonging to the protoilludane, illudalane, and botryane sesquiterpenoid classes. These compound classes represent indeed promising targets for further development, as in the case of the melleolides produced by *Armillaria* spp. (Agaricales, Basidiomycota) and the anticancer lead compounds illudins from the genus *Omphalotus* (Agaricales, Basidiomycota) [75, 76].

In summary, this study developed a stepwise methodology for formulating culture media tailored to *Diaporthe caliensis*. By integrating metabolomic profiling, we gained insights into the chemical diversity of this fungus and its relationship with factors like carbon and nitrogen source concentrations, type of nitrogen source, supplemental solutions, and pH regulation. Among our findings, it is noted that the addition of micronutrients and the presence of buffer significantly influenced the production of polyketide-lactones related to phomol, setting the path towards future optimization of its production. In fact, this methodology not only facilitates the targeted discovery of novel molecules but also highlights the potential of this fungus for producing metabolites from distinct compound classes while maintaining desirable biological properties, which targeted purification, will be the endeavor of future studies.

## Conclusions

This study presents a practical approach for designing tailored media based on nutritional limitations to enhance the production of antimicrobial SMs during the submerged fermentation of *Diaporthe caliensis*. By systematically analyzing key bioprocess parameters, we evaluated both metabolomic changes and biological activities in response to nutritional adjustments. This demonstrated that the biosynthetic potential of the studied fungus could be further explored by inducing specific nutritional stress conditions. Notably, our findings revealed that the antibacterial effects observed across different treatments are not solely dependent on the production of phomol-like molecules, which were only significantly promoted in a limited number of treatments. This indicates that *Diaporthe caliensis* has the ability to produce a diverse range of antimicrobial compounds under varying conditions. Consequently, our study highlights the need for future research aimed at enhancing our understanding of the chemical and biosynthetic diversity of this rather unexplored fungal biofactory, which could be employed for the sustainable production of novel antimicrobial agents.

## Electronic supplementary material

Below is the link to the electronic supplementary material.

Supplementary Material 1

Supplementary Material 2

Supplementary Material 3

Supplementary Material 4

Supplementary Material 5

## Data Availability

Data is provided within the manuscript or supplementary information files.

## References

[1] Sharrar AM, Crits-Christoph A, Méheust R, Diamond S, Starr EP, Banfield JF. Bacterial secondary metabolite biosynthetic potential in soil varies with phylum, depth, and vegetation type. Appl Environ Microbiol. 2020;11. 10.1128/mBio.00416-20 PMC7298704 32546614

[2] Singh BP, Rateb ME, Rodriguez-Couto S, De Moraes Polizeli LT, Li M, Editorial WJ. Microbial secondary metabolites: recent developments and technological challenges. Front Microbiol. 2019;10. 10.3389/fmicb.2019.00914 PMC6498875 31105684

[3] Keller NP. Fungal secondary metabolism: regulation, function and drug discovery. Nat Rev Microbiol. 2019;17:167–80. 30531948 10.1038/s41579-018-0121-1 PMC6381595

[4] Guerriero G, Berni R, Muñoz-Sanchez JA, Apone F, Abdel-Salam EM, Qahtan AA et al. Production of plant secondary metabolites: examples, tips and suggestions for biotechnologists. Genes (Basel). 2018;6:9. 10.3390/genes9060309 PMC6027220 29925808

[5] Teoh ES. Secondary Metabolites of Plants. Medicinal Orchids of Asia. 2016. pp. 59–73.

[6] Seyedsayamdost MR. Toward a global picture of bacterial secondary metabolism. J Ind Microbiol Biotechnol. 2019;46:301–11. 30684124 10.1007/s10295-019-02136-y PMC6779422

[7] Santamaria G, Liao C, Lindberg C, Chen Y, Wang Z, Rhee K et al. Evolution and regulation of microbial secondary metabolism. Elife. 2022;11. 10.7554/eLife.76119 PMC9708071 36409069

[8] Liu Y, Bastiaan-Net S, Wichers HJ. Current understanding of the structure and function of Fungal Immunomodulatory proteins. Front Nutr. 2020;7. 10.3389/fnut.2020.00132 PMC7461872 33015115

[9] Ramírez-Villalobos JM, Gomez-Flores R, Velázquez-Flores PV, Morán-Santibáñez KS, Tamez-Guerra P, Pérez-González O et al. Effect of Culture conditions of Lophocereus Marginatus Endophytic Fungi on yield and anticancer and antioxidant activities. Int J Environ Res Public Health. 2023;20. 10.3390/ijerph20053948 PMC10001847 36900961

[10] Kousar R, Naeem M, Jamaludin MI, Arshad A, Shamsuri AN, Ansari N et al. Exploring the anticancer activities of novel bioactive compounds derived from endophytic fungi: mechanisms of action, current challenges and future perspectives. Am J Cancer Res. 2022;12. PMC9360238 35968347

[11] Fasinu PS, Okoye FBC, Abiodun OO, Kamdem RST, Ogbole OO. Editorial: fungal bioactive metabolites of pharmacological relevance. Front Pharmacol. 2022;13. 10.3389/fphar.2022.912068 PMC9213805 35754512

[12] Jakubczyk D, Dussart F. Selected fungal natural products with antimicrobial properties. Molecules. 2020;25. 10.3390/molecules25040911 PMC7070998 32085562

[13] Atanasov AG, Zotchev SB, Dirsch VM, Orhan IE, Banach M, Rollinger JM, et al. Natural products in drug discovery: advances and opportunities. Nat Rev Drug Discov. 2021;20:200–16. 33510482 10.1038/s41573-020-00114-z PMC7841765

[14] Craney A, Ahmed S, Nodwell J. Towards a new science of secondary metabolism. J Antibiot. 2013;66:387–400. 10.1038/ja.2013.25 23612726

[15] Yang X, Wu P, Xue J, Li H, Wei X. Cytochalasans from endophytic fungus *Diaporthe sp.* SC-J0138. Fitoterapia. 2020;145. 10.1016/j.fitote.2020.104611 32437736

[16] Yu JJ, Yang HX, Zhang FL, He J, Li ZH, Liu JK, et al. Secondary metabolites from cultures of the kiwi-associated fungus *Diaporthe phragmitis* and their antibacterial activity assessment. Phytochem Lett. 2021;46:143–8.

[17] Patil RH, Patil MP, Maheshwari VL. Bioactive secondary metabolites from endophytic Fungi: a review of Biotechnological Production and their potential applications. Stud Nat Prod Chem. 2016;49:189–205.

[18] Srivastava N, Srivastava M, Ramteke PW, Mishra PK. Solid-state fermentation strategy for microbial metabolites production: An overview. New and Future Developments in Microbial Biotechnology and Bioengineering: Microbial Secondary Metabolites Biochemistry and Applications. 2019. pp. 345–54.

[19] Webb C. Design aspects of Solid State Fermentation as Applied to Microbial Bioprocessing. J Appl Biotechnol Bioeng. 2017;4.

[20] Aidoo R, Kwofie EM, Adewale P, Lam E, Ngadi M. Overview of single cell protein: production pathway, sustainability outlook, and digital twin potentials. Trends Food Sci Technol. 2023;138:577–98.

[21] Zhao YS, Eweys AS, Zhang JY, Zhu Y, Bai J, Darwesh OM et al. Fermentation affects the antioxidant activity of plant-based food material through the release and production of bioactive components. Antioxidants. 2021;10. 10.3390/antiox10122004 PMC8698425 34943107

[22] Bode HB, Bethe B, Höfs R, Zeeck A. Big effects from small changes: possible ways to explore nature’s chemical diversity. ChemBioChem. 2002;3:619–27. 12324995 10.1002/1439-7633(20020703)3:7<619::AID-CBIC619>3.0.CO;2-9

[23] Rodríguez Martín-Aragón V, Trigal Martínez M, Cuadrado C, Daranas AH, Fernández Medarde A. Sánchez López JM. OSMAC Approach and Cocultivation for the induction of secondary metabolism of the Fungus *Pleotrichocladium opacum*. ACS Omega. 2023;8. 10.1021/acsomega.3c06299 PMC10601420 37901491

[24] Meena MG, Lane MJ, Tannous J, Carrell AA, Abraham PE, Giannone RJ et al. A glimpse into the fungal metabolomic abyss: novel network analysis reveals relationships between exogenous compounds and their outputs. PNAS Nexus. 2023;2. 10.1093/pnasnexus/pgad322 PMC10581544 37854706

[25] Kottmeier K, Müller C, Huber R, Büchs J. Increased product formation induced by a directed secondary substrate limitation in a batch *Hansenula polymorpha* culture. Appl Microbiol Biotechnol. 2010;86:93–101. 19859706 10.1007/s00253-009-2285-0

[26] Youngquist JT, Rose JP, Pfleger BF. Free fatty acid production in *Escherichia coli* under phosphate-limited conditions. Appl Microbiol Biotechnol. 2013;97. 10.1007/s00253-013-4911-0 23619909

[27] Azzollini A, Boggia L, Boccard J, Sgorbini B, Lecoultre N, Allard PM et al. Dynamics of metabolite induction in fungal co-cultures by metabolomics at both volatile and non-volatile levels. Front Microbiol. 2018;9. 10.3389/fmicb.2018.00072 PMC5807337 29459851

[28] Lv G, Xu Y, Tu Y, Cheng X, Zeng B, Huang J et al. Effects of Nitrogen and Phosphorus limitation on fatty acid contents in *aspergillus oryzae*. Front Microbiol. 2021;12. 10.3389/fmicb.2021.739569 PMC8566876 34745041

[29] Rodríguez-Torres M, Romo-Buchelly J, Orozco-Sánchez F. Effects of oxygen transfer rate on the L(+) lactic acid production by *Rhizopus Oryzae* NRRL 395 in stirred tank bioreactor. Biochem Eng J. 2022;187.

[30] Dinger R, Lattermann C, Flitsch D, Fischer JP, Kosfeld U, Büchs J. Device for respiration activity measurement enables the determination of oxygen transfer rates of microbial cultures in shaken 96-deepwell microtiter plates. Biotechnol Bioeng. 2022;119. 10.1002/bit.28022 34951007

[31] Jiang L, Ma Q, Li A, Sun R, Tang G, Huang X et al. Bioactive secondary metabolites produced by fungi of the genus *Diaporthe (Phomopsis)*: structures, biological activities, and biosynthesis. Arab J Chem. 2023;16.

[32] Liu X, Locasale JW, Metabolomics. A primer. Trends Biochem Sci. 2017;42:274–84. 28196646 10.1016/j.tibs.2017.01.004 PMC5376220

[33] Hoyos LV, Chaves A, Grandezz D, Medina A, Correa J, Ramirez-Castrillon M, et al. Systematic screening strategy for fungal laccase activity of endophytes from *Otoba gracilipes* with bioremediation potential. Fungal Biol. 2023;127:1298–311. 37821152 10.1016/j.funbio.2023.08.003

[34] Mendez MJ, Caicedo NH, Salamanca C. *Trametes Elegans*: a fungal endophytic isolate from *Otoba gracilipes* as biocatalyst for natural flavors production. N Biotechnol. 2018;44.

[35] Caicedo NH, Davalos AF, Puente PA, Rodríguez AY, Caicedo PA. Antioxidant activity of exo-metabolites produced by *Fusarium oxysporum*: an endophytic fungus isolated from leaves of *Otoba gracilipes*. Microbiologyopen. 2019;8. 10.1002/mbo3.903 PMC6813440 31297981

[36] Charria-Girón E, Espinosa MC, Zapata-Montoya A, Méndez MJ, Caicedo JP, Dávalos AF et al. Evaluation of the antibacterial activity of crude extracts obtained from cultivation of native endophytic Fungi belonging to a Tropical Montane Rainforest in Colombia. Front Microbiol. 2021;12. 10.3389/fmicb.2021.716523 PMC8485978 34603244

[37] Charria-Girón E, Marin-Felix Y, Beutling U, Franke R, Brönstrup M, Vasco-Palacios AM et al. Metabolomics insights into the polyketide-lactones produced by *Diaporthe caliensis* sp. nov., an endophyte of the medicinal plant *Otoba gracilipes*. Pidot SJ, editor. Microbiol Spectr. 2023. 10.1128/spectrum.02743-23 PMC10715209 37921483

[38] Dolecek C, Shakoor S, Basnyat B, Okwor T, Sartorius B. Drug-resistant bacterial infections: we need urgent action and investment that focus on the weakest link. PLoS Biol. 2022;20. 10.1371/journal.pbio.3001903 PMC9710749 36383561

[39] Salam MA, Al-Amin MY, Salam MT, Pawar JS, Akhter N, Rabaan AA, et al. Antimicrobial Resistance: a growing serious threat for Global Public Health. Healthcare (Switzerland; 2023. 10.3390/healthcare11131946 PMC10340576 37444780

[40] Tang KWK, Millar BC, Moore JE. Antimicrobial Resistance (AMR). Br J Biomed Sci. 2023;80 10.3389/bjbs.2023.11387 PMC10336207 37448857

[41] Duetz WA, Witholt B. Oxygen transfer by orbital shaking of square vessels and deepwell microtiter plates of various dimensions. Biochem Eng J. 2004;17:181–5.

[42] Philip P, Meier K, Kern D, Goldmanns J, Stockmeier F, Bähr C et al. Systematic evaluation of characteristics of the membrane-based fed-batch shake flask. Microb Cell Fact. 2017;16. 10.1186/s12934-017-0741-6 PMC5514527 28716035

[43] Sáez-Plaza P, Navas MJ, Wybraniec S, Michałowski T, Asuero AG. An overview of the Kjeldahl Method of Nitrogen Determination. Part II. Sample Preparation, Working Scale, Instrumental Finish, and Quality Control. Crit Rev Anal Chem. 2013;43.

[44] Sluiter A, Hames B, Ruiz R, Scarlata C, Sluiter J, Templeton D. Determination of structural carbohydrates and lignin in biomass determination of structural carbohydrates and lignin in biomass. National Renewable Energy Laboratory (NREL); 2010.

[45] Harms K, Surup F, Stadler M, Stchigel AM, Marin-felix Y. Morinagadepsin, a depsipeptide from the fungus morinagamyces vermicularis gen. Et comb nov Microorganisms. 2021;9. 10.3390/microorganisms9061191 PMC8230337 34073017

[46] Kemkuignou B, Lambert C, Stadler M, Kouam Fogue S, Marin-Felix Y. Unprecedented antimicrobial and cytotoxic polyketides from cultures of *Diaporthe Africana* sp. nov. J Fungi. 2023;9:781. 10.3390/jof9070781 PMC10381184 37504769

[47] Kemkuignou B, Schweizer L, Lambert C, Anoumedem EGM, Kouam SF, Stadler M, et al. New polyketides from the liquid culture of *Diaporthebreyniae sp.* nov. (*Diaporthales, Diaporthaceae*). MycoKeys. 2022;90:85–118. 36760420 10.3897/mycokeys.90.82871 PMC9849082

[48] Pfütze S, Khamsim A, Surup F, Decock C, Matasyoh JC, Stadler M. Calamene-type Sesqui-, Mero-, and Bis-sesquiterpenoids from cultures of *Heimiomyces sp*., a Basidiomycete Collected in Africa. J Nat Prod. 2023;86. 10.1021/acs.jnatprod.2c01015 PMC9972471 36779910

[49] de Souza ARC, Baldoni DB, Lima J, Porto V, Marcuz C, Ferraz RC et al. Bioherbicide production by *Diaporthe sp.* isolated from the Brazilian pampa biome. Biocatal Agric Biotechnol. 2015;4.

[50] Gong Z, Zhang S, Liu J. Recent Advances in Chitin Biosynthesis Associated with the morphology and secondary Metabolite synthesis of Filamentous Fungi in Submerged Fermentation. J Fungi. 2023;9. 10.3390/jof9020205 PMC9967639 36836319

[51] Papagianni M. Fungal morphology and metabolite production in submerged mycelial processes. Biotechnol Adv. 2004;22. 10.1016/j.biotechadv.2003.09.005 14665401

[52] Van Santen JA, Jacob G, Singh AL, Aniebok V, Balunas MJ, Bunsko D et al. The Natural products Atlas: an Open Access Knowledge Base for Microbial Natural products Discovery. ACS Cent Sci. 2019;5. 10.1021/acscentsci.9b00806 PMC6891855 31807684

[53] Ruttkies C, Schymanski EL, Wolf S, Hollender J, Neumann S. MetFrag relaunched: incorporating strategies beyond in silico fragmentation. J Cheminform. 2016;8. 10.1186/s13321-016-0115-9 PMC4732001 26834843

[54] Anderlei T, Büchs J. Device for sterile online measurement of the oxygen transfer rate in shaking flasks. Biochem Eng J. 2001;7. 10.1016/s1369-703x(00)00116-9 11173305

[55] Dührkop K, Nothias LF, Fleischauer M, Reher R, Ludwig M, Hoffmann MA et al. Systematic classification of unknown metabolites using high-resolution fragmentation mass spectra. Nat Biotechnol. 2021;39. 10.1038/s41587-020-0740-8 33230292

[56] Drew SW, Demain AL. Effect of primary metabolites on secondary metabolism. Annu Rev Microbiol. 1977;31:343–56. 71875 10.1146/annurev.mi.31.100177.002015

[57] Sánchez S, Chávez A, Forero A, García-Huante Y, Romero A, Sánchez M, et al. Carbon source regulation of antibiotic production. J Antibiot (Tokyo). 2010;63:442–59. 20664603 10.1038/ja.2010.78

[58] Ruiz B, Chávez A, Forero A, García-Huante Y, Romero A, Snchez M et al. Production of microbial secondary metabolites: regulation by the carbon source. Crit Rev Microbiol. 2010;36. 10.3109/10408410903489576 20210692

[59] Tudzynski B. Nitrogen regulation of fungal secondary metabolism in fungi. Front Microbiol. 2014;5. 10.3389/fmicb.2014.00656 PMC4246892 25506342

[60] Ehrlich KC, Cotty PJ. Variability in nitrogen regulation of aflatoxin production by *aspergillus flavus* strains. Appl Microbiol Biotechnol. 2002;60. 10.1007/s00253-002-1094-5 12382060

[61] Abbas A, Valez H, Dobson ADW. Analysis of the effect of nutritional factors on OTA and OTB biosynthesis and polyketide synthase gene expression in *aspergillus ochraceus*. Int J Food Microbiol. 2009;135. 10.1016/j.ijfoodmicro.2009.07.014 19682762

[62] Scherlach K, Sarkar A, Schroeckh V, Dahse HM, Roth M, Brakhage AA et al. Two Induced Fungal Polyketide pathways converge into Antiproliferative Spiroanthrones. ChemBioChem. 2011;12. 10.1002/cbic.201100132 21698737

[63] Liu X, Wang X, Xu J, Xia J, Lv J, Zhang T et al. Citric acid production by *Yarrowia lipolytica* SWJ-1b using corn steep liquor as a source of organic nitrogen and vitamins. Ind Crops Prod. 2015;78.

[64] Marzluf GA. Genetic regulation of nitrogen metabolism in the fungi. Microbiol Mol Biol Rev. 1997;61. 10.1128/mmbr.61.1.17-32.1997 PMC232598 9106362

[65] Marzluf GA. Regulation of sulfur and nitrogen metabolism in filamentous fungi. Annu Rev Microbiol. 1993;47. 10.1146/annurev.mi.47.100193.000335 8257101

[66] Rózsa M, Măniuțiu D-N, Egyed E. Influence of Magnesium (mg) source on the *cordyceps militaris* (L.) Mushroom Mycelium Growth. Curr Trends Nat Sci. 2021;10.

[67] Philpott CC. Iron uptake in fungi: a system for every source. Biochim Biophys Acta Mol Cell Res. 2006;1763. 10.1016/j.bbamcr.2006.05.008 16806534

[68] Robinson JR, Isikhuemhen OS, Anike FN. Fungal–metal interactions: a review of toxicity and homeostasis. J Fungi. 2021;7. 10.3390/jof7030225 PMC8003315 33803838

[69] Rodríguez-Navarro A, Benito B. Sodium or potassium efflux ATPase. A fungal, bryophyte, and protozoal ATPase. Biochim Biophys Acta Biomembr. 2010;1798. 10.1016/j.bbamem.2010.07.009 20650263

[70] Khademi Z, Heravi MM. Applications of Claisen condensations in total synthesis of natural products. An old reaction, a new perspective. Tetrahedron. 2022;103.

[71] Xie S, Wu Y, Qiao Y, Guo Y, Wang J, Hu Z et al. Protoilludane, Illudalane, and Botryane Sesquiterpenoids from the endophytic fungus *phomopsis sp.* TJ507A. J Nat Prod. 2018;81. 10.1021/acs.jnatprod.7b00889 29771527

[72] Lin T, Wang GH, Lin X, Hu ZY, Chen QC, Xu Y et al. Three new oblongolides from *Phomopsis sp.* XZ-01, an endophytic fungus from Camptotheca acuminate. Molecules. 2011;16. 10.3390/molecules16043351 PMC6260605 21512443

[73] Li LM, Zou Q, Li GY. Chromones from an ascomycete, Chaetomium Aureus. Chin Chem Lett. 2010;21.

[74] Li H, Tian JM, Tang HY, Pan SY, Zhang AL, Gao JM. Chaetosemins A-E, new chromones isolated from an Ascomycete *Chaetomium Seminudum* and their biological activities. RSC Adv. 2015;5.

[75] Chaverra-Muñoz L, Briem T, Hüttel S. Optimization of the production process for the anticancer lead compound illudin M: improving titers in shake-flasks. Microb Cell Fact. 2022;21. 10.1186/s12934-022-01827-z PMC9148526 35643529

[76] Pfütze S, Charria-Girón E, Schulzke E, Toshe R, Khonsanit A, Franke R et al. Depicting the Chemical diversity of Bioactive Meroterpenoids produced by the largest organism on Earth. Angew Chem Int Ed. 2024;63. 10.1002/anie.202318505 38390787

