# Supplementary material for: Tailored culture strategies to promote antimicrobial secondary metabolite production in Diaporthe caliensis: a metabolomic approach
Source: Microb Cell Fact. 2024 Dec 5;23:328. doi: 10.1186/s12934-024-02567-y (PMC11619134; doi:10.1186/s12934-024-02567-y)
Supplement: Supplementary file 1 — Supplementary Material 1 [file 12934_2024_2567_MOESM1_ESM.docx]

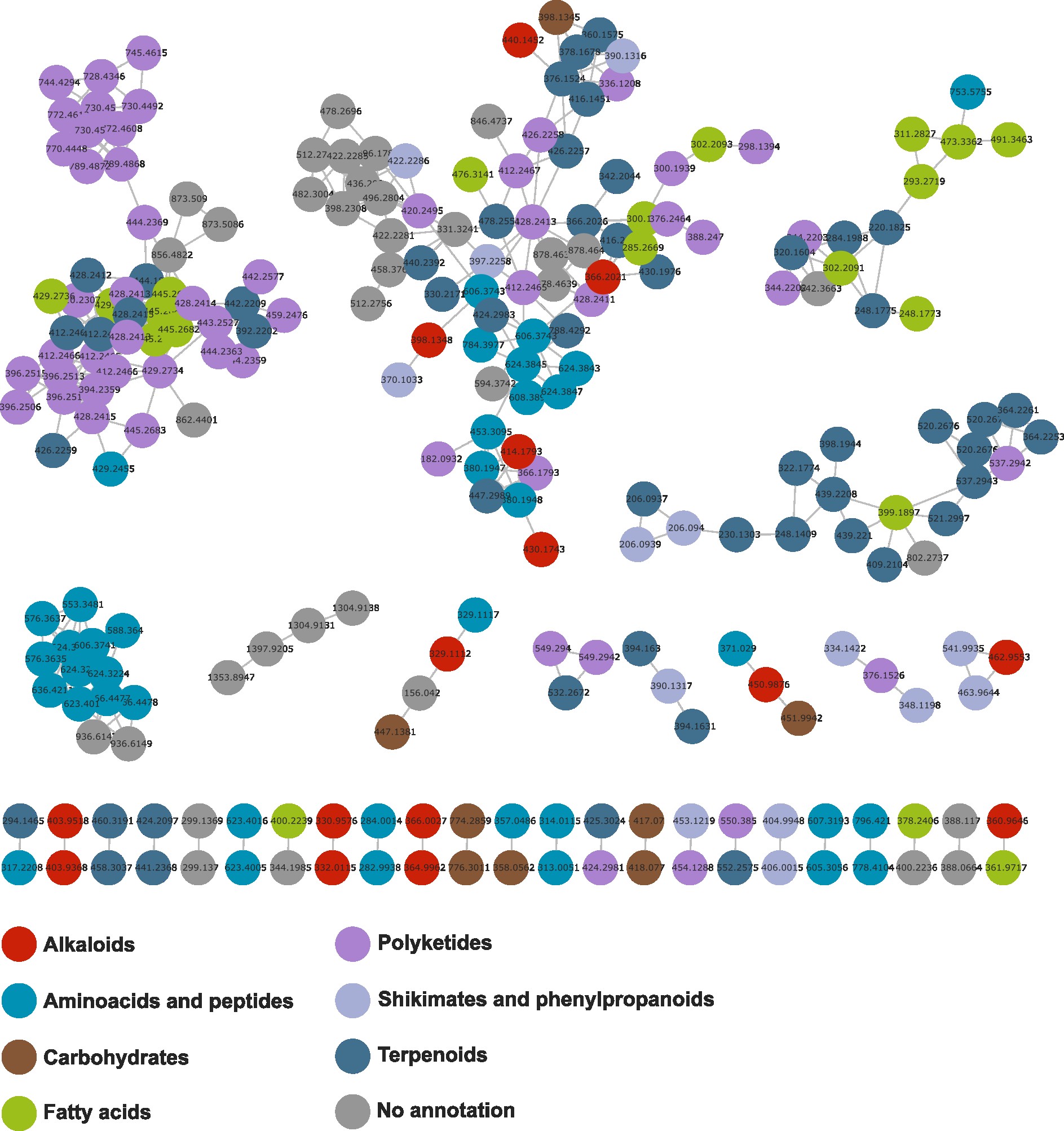


**Fig. S1.** Feature Based Molecular Networking (FBMN) of compounds detected in all treatments. Nodes are colored according to the most specific natural product pathway as obtained from CANPOPUS analysis.
