# Supplementary material for: Tailored culture strategies to promote antimicrobial secondary metabolite production in Diaporthe caliensis: a metabolomic approach
Source: Microb Cell Fact. 2024 Dec 5;23:328. doi: 10.1186/s12934-024-02567-y (PMC11619134; doi:10.1186/s12934-024-02567-y)
Supplement: Supplementary file 2 — Supplementary Material 2 [file 12934_2024_2567_MOESM2_ESM.docx]

**Table S1.** Dereplicated metabolites from the cultures of *Diaporthe caliensis*.

| **Name** | **rt** | ***m/z*** | **Formula** | **Annotation*** | **Database** |
| --- | --- | --- | --- | --- | --- |
| (13R)-diaporphthalide A | 5.7 | 297.0956 | C_14_H_16_O_7_ | Level 2 | NP Atlas (Genus: *Diaporthe*) |
| (1R,2E,4S,5R)-1-[(2R)-5-oxotetrahydrofuran-2-yl]-4,5-dihydroxy-hex-2-en-1-yl(2E)-2-methylbut-2-enoate | 9.74 | 321.1312 | C_15_H_22_O_6_ | Level 2 | NP Atlas (Genus: *Diaporthe*) |
| 1-O-methylterricolyne | 7.84 | 207.1011 | C_12_H_14_O_3_ | Level 2 | NP Atlas (Genus: *Diaporthe*) |
| 2,3-Dihydromycoepoxydiene | 8.02 | 315.1202 | C_16_H_20_O_5_ | Level 2 | NP Atlas (Genus: *Phomopsis*) |
| 3a,9a-deoxy-3a-hydroxy-1-dehydroxyarthrinone | 7 | 267.0862 | C_13_H_14_O_6_ | Level 2 | NP Atlas (Genus: *Diaporthe*) |
| Carneic acid D | 12.86 | 313.2162 | C_21_H_30_O_3_ | Level 2 | NP Atlas (Genus: *Diaporthe*) |
| Cerdarin | 5.65 | 277.0706 | C_14_H_12_O_6_ | Level 2 | NP Atlas (Genus: *Diaporthe*) |
| Cerdarin | 6.41 | 277.0705 | C_14_H_12_O_6_ | Level 2 | NP Atlas (Genus: *Diaporthe*) |
| Chaetoaurin | 10.67 | 335.1491 | C_18_H_22_O_6_ | Level 2 | NP Atlas (Genus: *Diaporthe*) |
| Chaetolactone | 11.19 | 253.1433 | C_14_H_20_O_4_ | Level 2 | NP Atlas (Genus: *Diaporthe*) |
| Chaetolactone | 11.42 | 253.1434 | C_14_H_20_O_4_ | Level 2 | NP Atlas (Genus: *Diaporthe*) |
| Chaetolactone | 12.22 | 253.1433 | C_14_H_20_O_4_ | Level 2 | NP Atlas (Genus: *Diaporthe*) |
| Chaetolactone | 12.6 | 253.1434 | C_14_H_20_O_4_ | Level 2 | NP Atlas (Genus: *Diaporthe*) |
| Chaetolactone | 13.97 | 253.1433 | C_14_H_20_O_4_ | Level 2 | NP Atlas (Genus: *Diaporthe*) |
| Diaporol C | 11.47 | 239.2003 | C_15_H_28_O_3_ | Level 2 | NP Atlas (Genus: *Diaporthe*) |
| Diaporol C | 13.82 | 239.2004 | C_15_H_28_O_3_ | Level 2 | NP Atlas (Genus: *Diaporthe*) |
| Diaporol C | 13.83 | 257.2111 | C_15_H_28_O_3_ | Level 2 | NP Atlas (Genus: *Diaporthe*) |
| Diaporol H | 10.72 | 235.1692 | C_15_H_24_O_3_ | Level 2 | NP Atlas (Genus: *Diaporthe*) |
| Dothiorelone J | 10.18 | 333.131 | C_16_H_22_O_6_ | Level 2 | NP Atlas (Genus: *Phomopsis*) |
| EI-1941-2 | 12.42 | 221.0807 | C_12_H_14_O_5_ | Level 2 | NP Atlas (Genus: *Diaporthe*) |
| Longiphthalidin A | 6.66 | 253.0704 | C_12_H_12_O_6_ | Level 2 | NP Atlas (Genus: *Diaporthe*) |
| Oblongolide F | 10.02 | 237.1483 | C_14_H_20_O_3_ | Level 2 | NP Atlas (Genus: *Phomopsis*) |
| Oblongolide O | 13.63 | 237.1483 | C_14_H_20_O_3_ | Level 2 | NP Atlas (Genus: *Phomopsis*) |
| Oblongolide P | 8.59 | 237.1483 | C_14_H_20_O_3_ | Level 2 | NP Atlas (Genus: *Phomopsis*) |
| Oblongolide Q | 13.87 | 237.1482 | C_14_H_20_O_3_ | Level 2 | NP Atlas (Genus: *Phomopsis*) |
| Phomodiol | 13.82 | 249.1848 | C_16_H_26_O_3_ | Level 2 | NP Atlas (Genus: *Phomopsis*) |
| Phomodiol | 14.05 | 249.1847 | C_16_H_26_O_3_ | Level 2 | NP Atlas (Genus: *Phomopsis*) |
| Phomol | 13.96 | 395.2431 | C_22_H_36_O_7_ | Level 1 | In house library |
| Phomolactone C | 7.05 | 223.0957 | C_12_H_14_O_4_ | Level 2 | NP Atlas (Genus: *Phomopsis*) |
| Phomolide A | 7.27 | 209.1169 | C_12_H_16_O_3_ | Level 2 | NP Atlas (Genus: *Phomopsis*) |
| Phomolide B | 9.35 | 209.1168 | C_12_H_18_O_4_ | Level 2 | NP Atlas (Genus: *Phomopsis*) |
| Phomolide D | 8.54 | 227.1276 | C_12_H_20_O_5_ | Level 2 | NP Atlas (Genus: *Phomopsis*) |
| Phomophyllin C | 9.75 | 233.1534 | C_15_H_20_O_2_ | Level 2 | NP Atlas (Genus: *Phomopsis*) |
| Phomophyllin D | 9.1 | 231.1376 | C_15_H_20_O_3_ | Level 2 | NP Atlas (Genus: *Phomopsis*) |
| Phomophyllin E | 10.18 | 231.1377 | C_15_H_20_O_3_ | Level 2 | NP Atlas (Genus: *Phomopsis*) |
| Phomophyllin G | 12.68 | 233.1533 | C_15_H_20_O_2_ | Level 2 | NP Atlas (Genus: *Phomopsis*) |
| Phomoxydiene A | 10.52 | 307.1178 | C_16_H_18_O_6_ | Level 2 | NP Atlas (Genus: *Phomopsis*) |
| Spiroindicumide A | 9.06 | 335.185 | C_19_H_26_O_5_ | Level 2 | NP Atlas (Genus: *Diaporthe*) |
| Threo-L-2-[(2,4-dihydroxy-6-methylbenzoyl)oxy]-3-hydroxybutanoic acid | 6.72 | 293.0634 | C_12_H_14_O_7_ | Level 2 | NP Atlas (Genus: *Diaporthe*) |

*Level 1 correspond to annotations by comparison with in house standards; level 2 to putative annotations by comparison of measured MS/MS spectra and *in silico* predicted MS/MS spectra of compounds reported for *Diaporthe* and *Phomopsis* spp. in the NP Atlas. rt = retention time in min.


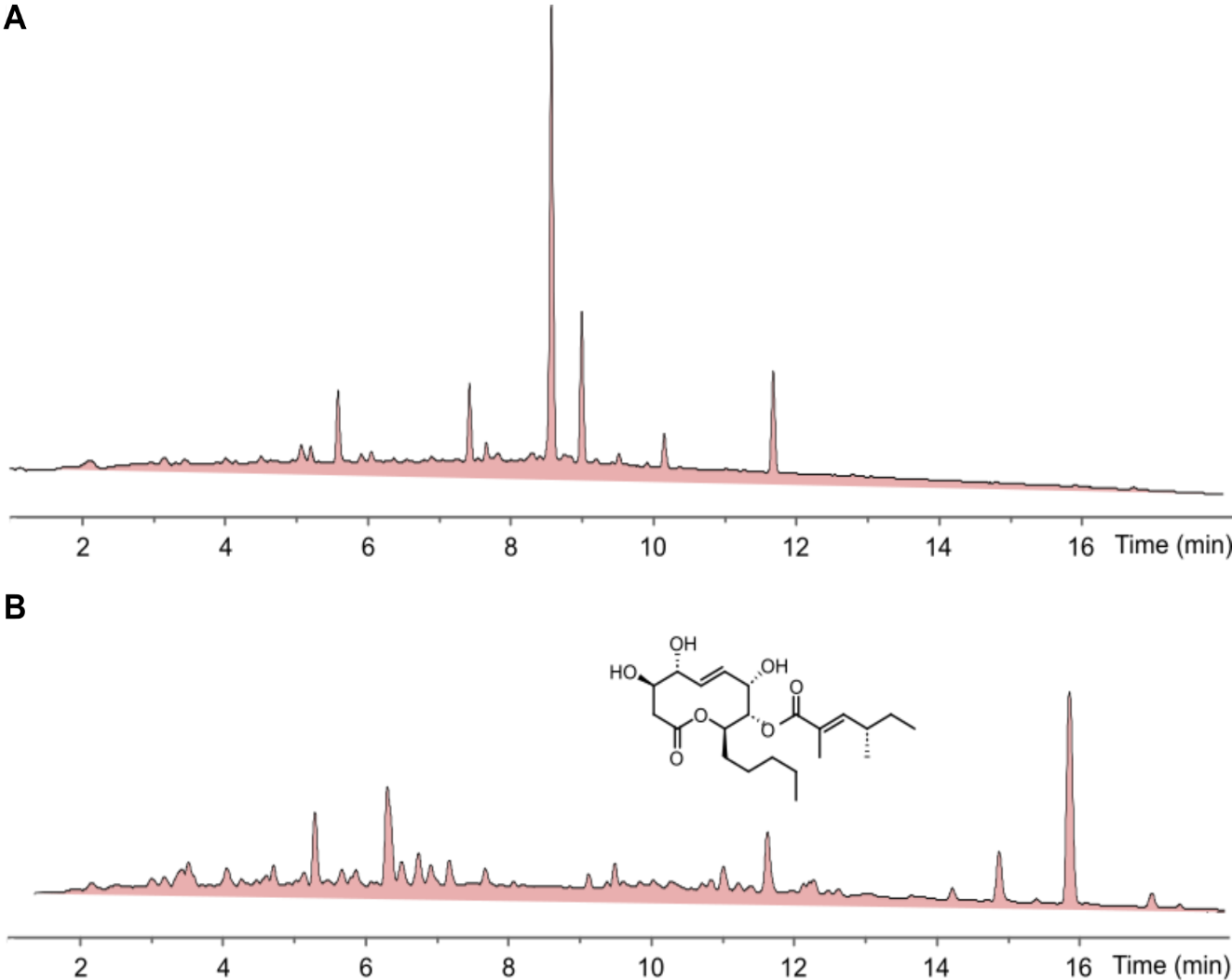


**Fig. S2.** HPLC-UV/Vis chromatograms at 210 nm of the crude extracts obtained from the cultivation of *Diaporthe caliensis* in the treatment C15-N0.75-L-M (B) (A) and in solid oat medium (B) as in the study from Charria-Girón et al. 2023.


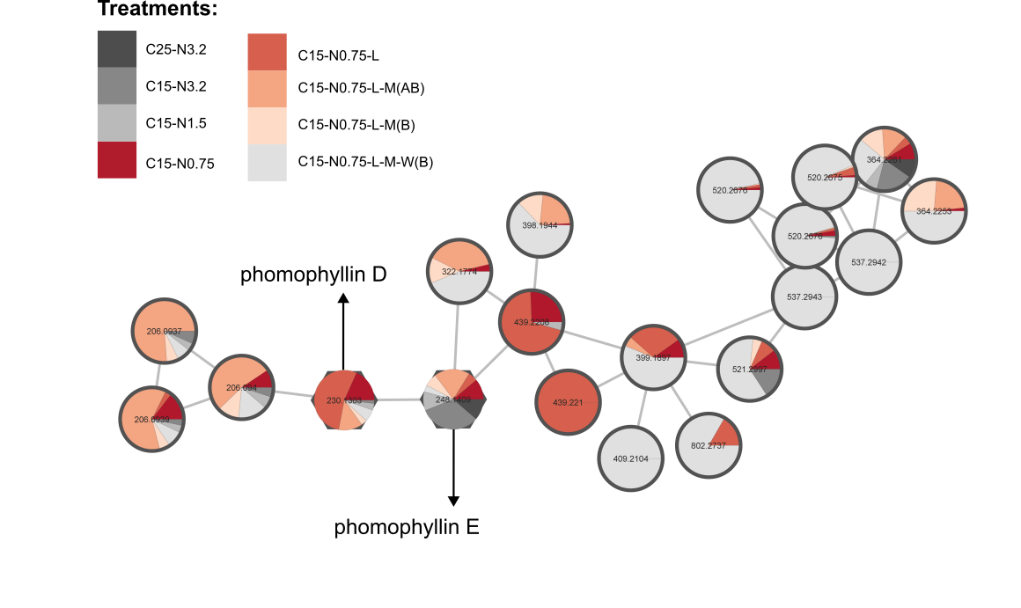


**Fig. S3.** Molecular family (MF) encompassing features putatively annotated as phomophyllin D and E.

**
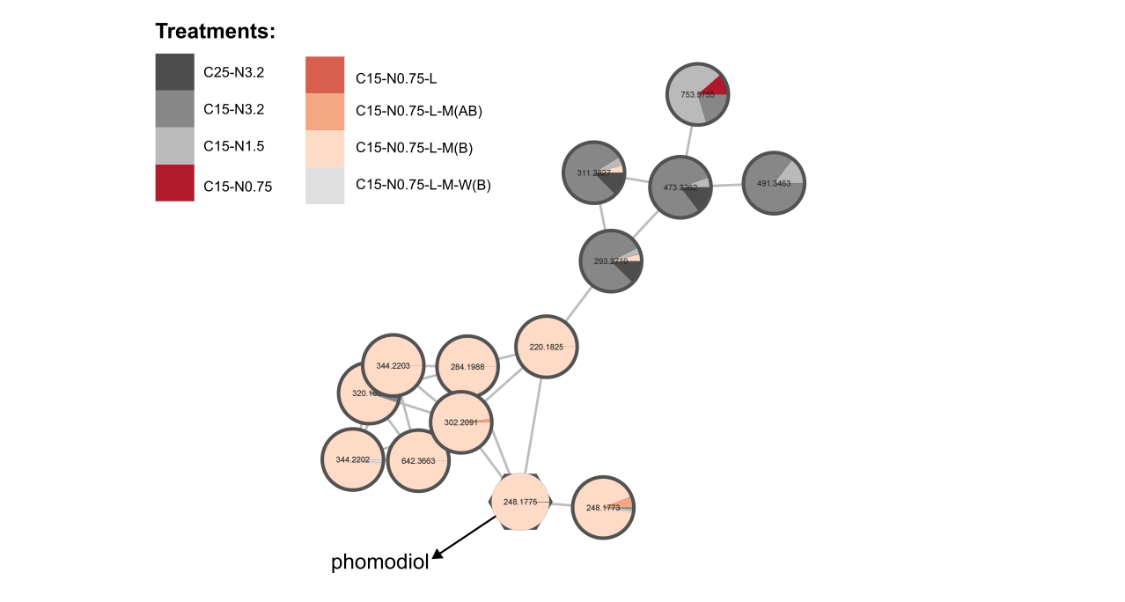
**

**Fig. S4.** Molecular family (MF) encompassing feature putatively annotated as phomodiol.

**
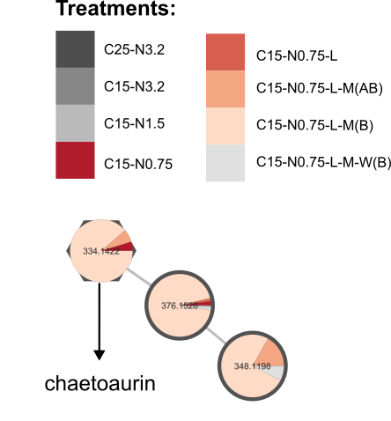
**

**Fig. S5.** Molecular family (MF) encompassing feature putatively annotated as chaetoaurin.
