# Supplementary material for: Tailored culture strategies to promote antimicrobial secondary metabolite production in Diaporthe caliensis: a metabolomic approach
Source: Microb Cell Fact. 2024 Dec 5;23:328. doi: 10.1186/s12934-024-02567-y (PMC11619134; doi:10.1186/s12934-024-02567-y)
Supplement: Supplementary file 3 — Supplementary Material 3 [file 12934_2024_2567_MOESM3_ESM.docx]

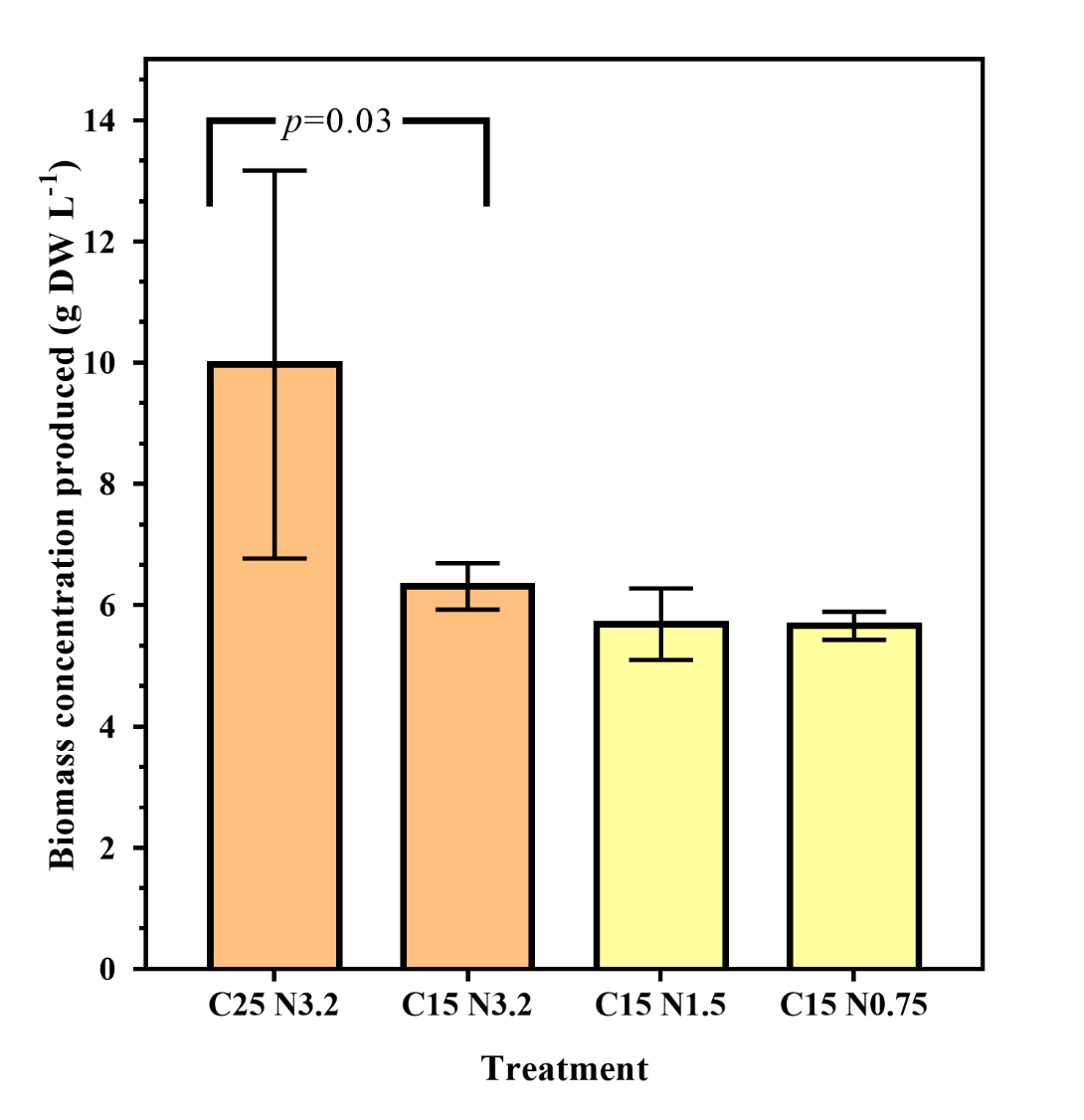


**Fig. S6**. Biomass concentration produced for four treatments in batch fermentation of *Diaporthe caliensis*.
