# Supplementary material for: Tailored culture strategies to promote antimicrobial secondary metabolite production in Diaporthe caliensis: a metabolomic approach
Source: Microb Cell Fact. 2024 Dec 5;23:328. doi: 10.1186/s12934-024-02567-y (PMC11619134; doi:10.1186/s12934-024-02567-y)
Supplement: Supplementary file 4 — Supplementary Material 4 [file 12934_2024_2567_MOESM4_ESM.docx]

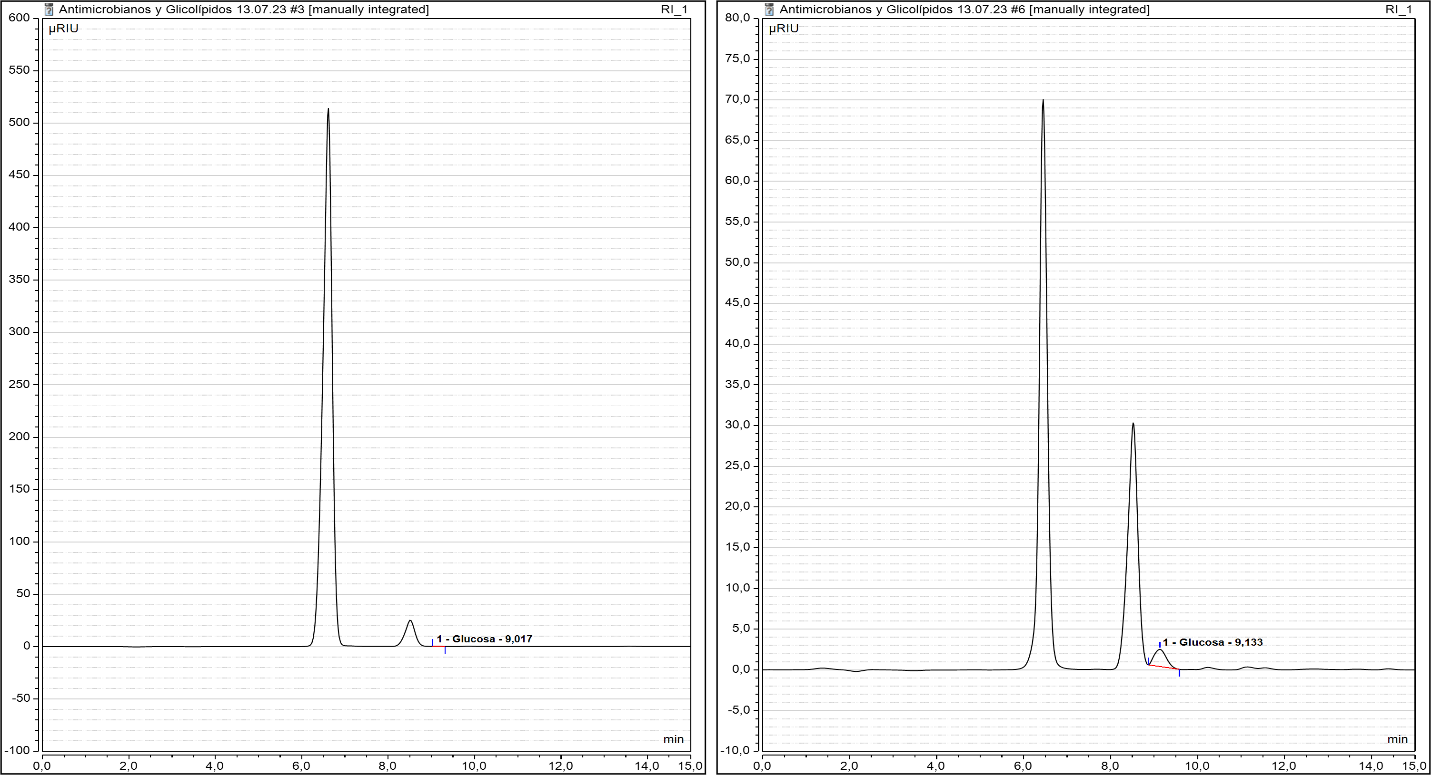
**Fig. S7**. Chromatograms obtained from HPLC analysis of residual sugars in the exhausted media of *Diaporthe caliensis* liquid cultures. “1” corresponds to glucose, the other peaks were not identified. Left: Exhausted medium corresponding to treatment C15-N3.2 **(carbon-limiting).** Right: Exhausted medium corresponding to treatment C15-N0.75 **(possible nitrogen limitation).**
